# Engineering Interfacial Built‐In Electric Fields via Work Function Matching Enables Photocoupled Osmotic Energy Recovery From Saline Dye Wastewater

**DOI:** 10.1002/smsc.70345

**Published:** 2026-07-23

**Authors:** Weixiang Tao, Peifang Wang, Gang Zhou, Yanhui Ao, Jie Wang, Yusuke Yamauchi

**Affiliations:** ^1^ Key Laboratory of Integrated Regulation and Resource Development on Shallow Lakes Ministry of Education College of Environment Hohai University Nanjing China; ^2^ Australian Institute for Bioengineering and Nanotechnology (AIBN) The University of Queensland Brisbane Queensland Australia‍; ^3^ Department of Materials Process Engineering, Graduate School of Engineering Nagoya University Nagoya Japan; ^4^ Department of Convergent Biotechnology and Advanced Materials Science Kyung Hee University Yongin‐si Gyeonggi‐do South Korea

**Keywords:** electric field, energy recovery, energy storage, energy transformation, environmental remediation, industrial wastewater treatment, ion transporter, membrane, power density, wastewater

## Abstract

Osmotic energy conversion offers a sustainable route to harvest electricity from salinity gradients. Beyond natural seawater/riverwater systems, saline waste streams rich in dissolved ions and persistent organic pollutants remain an underutilized resource for simultaneous energy recovery and remediation. Here, we report an asymmetric MoS_2_/oxygen‐doped ZIF‐8 membrane (MS‐ZIF‐A) that incorporates a built‐in electric field (IEF) to accelerate selective ion transport and enhance osmotic energy conversion. Work function matching between the functional layers optimizes interfacial band alignment, strengthening the internal driving force for ion migration and enabling a peak power density of 9.4 W m^−2^ under a 50‐fold NaCl salinity gradient. In simulated industrial wastewater containing NaCl and RhB, the membrane delivers power densities of up to 30 W m^−2^ while concurrently degrading organic pollutants. These results establish work function engineering as an effective strategy to construct interfacial IEF in asymmetric membranes, providing a general design principle for high‐performance blue‐energy devices and offering a promising route toward energy‐positive treatment of saline wastewater.

## Graphical abstract

An asymmetric MS‐ZIF‐A membrane with a work function‐regulated built‐in electric field enables directional ion transport across saline gradients while photocatalytically degrading organic pollutants. The system converts saline dye wastewater into a source for simultaneous energy recovery and water remediation.

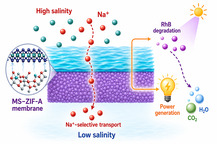

## 1 Introduction

As the global energy crisis intensifies, developing clean and renewable energy technologies has become increasingly urgent [1]. Salinity‐gradient energy, arising from the chemical potential difference between seawater and freshwater, offers an estimated global theoretical potential of ~2.4 TW [2, 3]. Beyond natural seawater/riverwater systems, many industrial waste streams contain high concentrations of dissolved ions together with persistent organic pollutants, representing an underexplored resource for electricity generation [4, 5]. If such polluted gradients can be harvested, energy‐intensive water treatment processes could be reconfigured into energy‐producing systems. Membrane‐based reverse electrodialysis (RED) has emerged as a scalable platform for converting ionic gradients into electrical power [6, 7]. To date, a wide range of advanced membranes, including two‐dimensional (2D) nanofluidic membranes, metal–organic framework (MOF) membranes, and covalent organic framework (COF) membranes, have been developed [8, 11]. Considerable effort has focused on engineering pore structures and surface charge densities to regulate ion transport within nanoscale channels [12, 13]. However, during operation, conventional membranes are often hindered by concentration polarization, which compromises ion selectivity and reduces energy conversion efficiency.

To mitigate concentration polarization and enhance directional ion transport, ion current rectification (ICR) has been introduced. Analogous to an ionic diode, ICR promotes ion transport in one direction while suppressing reverse transport, thereby increasing the net useful ion flux and reducing dissipative losses [14, 16]. As a result, asymmetric membranes with rectifying behavior can deliver higher power densities than single‐component membranes. Nevertheless, ionic diodes are typically achieved by combining heterogeneous materials that differ in pore structure, chemical compositions and surface charge polarity [17, 19], and the mechanistic origins of rectification in many hybrid systems remain incompletely understood. Consequently, rational materials pairing and interface design principles for high‐performance rectifying membranes in osmotic energy harvesting remain insufficiently developed.

From the standpoint of interfacial electrostatics, when two materials with different Fermi levels (*E*_f_) are brought into contact, interfacial electron redistribution occurs until thermodynamic equilibrium is reached, producing band bending and a built‐in electric field (IEF) across the junction. The IEF can strongly influence ion transport across the interface [20, 21]. Within this framework, electrons preferentially flow from the material with a lower work function (Φ) to that with a higher Φ, establishing an interfacial potential gradient [22]. Importantly, the orientation of this IEF relative to the ionic diffusion direction can either promote or impede ion migration. If the IEF opposes the net ion‐transport direction, ion migration is decelerated and selectivity is compromised. In contrast, when the IEF is aligned with ion diffusion, the interfacial driving force lowers the transport resistance and enhances selective ion flux. In semiconductor devices, for example, tuning Schottky barrier heights through a work‐function matching enables rectifying on ohmic contacts [23, 24]. Related concepts have also been extended to ionic systems: heterostructures such as VS_2_/VO_x_ can generate an IEF that lowers migration barriers and accelerates ion kinetics [25]. These observations suggest that deliberately constructing and modulating IEFs in asymmetric membranes could provide an effective route to robust ICR, offering a rational strategy to enhance ion transport and improve osmotic energy conversion.

In parallel, water pollution is becoming an escalating global challenge, driven in part by persistent organic contaminants such as synthetic dyes from textile effluents, which often resist conventional treatment processes and pose severe risks to aquatic ecosystems and human health [26, 27]. Photocatalysis offers a promising advanced oxidation strategy by converting light energy into reactive species (e.g., hydroxyl radicals, superoxide radicals) capable of mineralizing recalcitrant organics into benign products such as CO_2_ and H_2_O [28]. Importantly, polluted waters frequently contain coexisting metal ions; although these ions complicate treatment, they also provide an opportunity for synergistic integration of remediation and energy harvesting [29, 30]. In principle, photocatalytic degradation can alleviate organic fouling and maintain membrane activity, while dissolved ions serve as charge carriers for salinity gradient power generation [31]. Such an integrated approach could therefore enable simultaneous decontamination of complex wastewater and recovery of renewable energy.

Here we report an asymmetric membrane that integrates photocatalytic MoS_2_ with a ZIF‐8‐derived ion‐selective layer for photocoupled osmotic energy conversion and wastewater remediation. MoS_2_ and the ZIF‐based layer were selected because they provide complementary surface‐charge characteristics, distinct pore architectures, and favorable ion‐transport properties, thereby enabling the construction of an asymmetric nanofluidic interface. To elucidate the role of work function matching in regulating interfacial ion transport, the work function of ZIF‐8 was modulated by thermal treatment, increasing from 4.60 eV for pristine ZIF‐8 to 5.12 eV for oxygen‐doped ZIF‐A. This adjustment optimizes the band alignment with MoS_2_ and establishes a IEF oriented along the preferred cation‐transport direction, thereby reducing interfacial ion‐transport resistance and enhancing cation selectivity. Under a 50‐fold NaCl concentration gradient, the optimized MS‐ZIF‐A membrane delivered a peak power density of 9.4 W m^−2^, exceeding that of the MS‐ZIF membrane (5.9 W m^−2^). Furthermore, by leveraging the photocatalytic activity of MoS_2_, the membrane enables simultaneous osmotic energy harvesting and dye degradation under illumination. In saline dye wastewater, the MS‐ZIF‐A membrane achieved an output power density of up to 30 W m^−2^ while concurrently degrading RhB. Unlike conventional asymmetric membranes that primarily rely on pore geometry, surface‐charge polarity, or material composition to generate ion‐current rectification, this work introduces work function matching as an interfacial electronic structure engineering strategy to construct IEFs for accelerated ion transport. This study provides a rational design principle for high‐performance asymmetric membranes and offers an integrated route toward energy‐positive saline wastewater treatment.

## 2 Results and Discussion

### 2.1 Asymmetric Membrane Architecture for Efficient ICR

**FIGURE 1:**
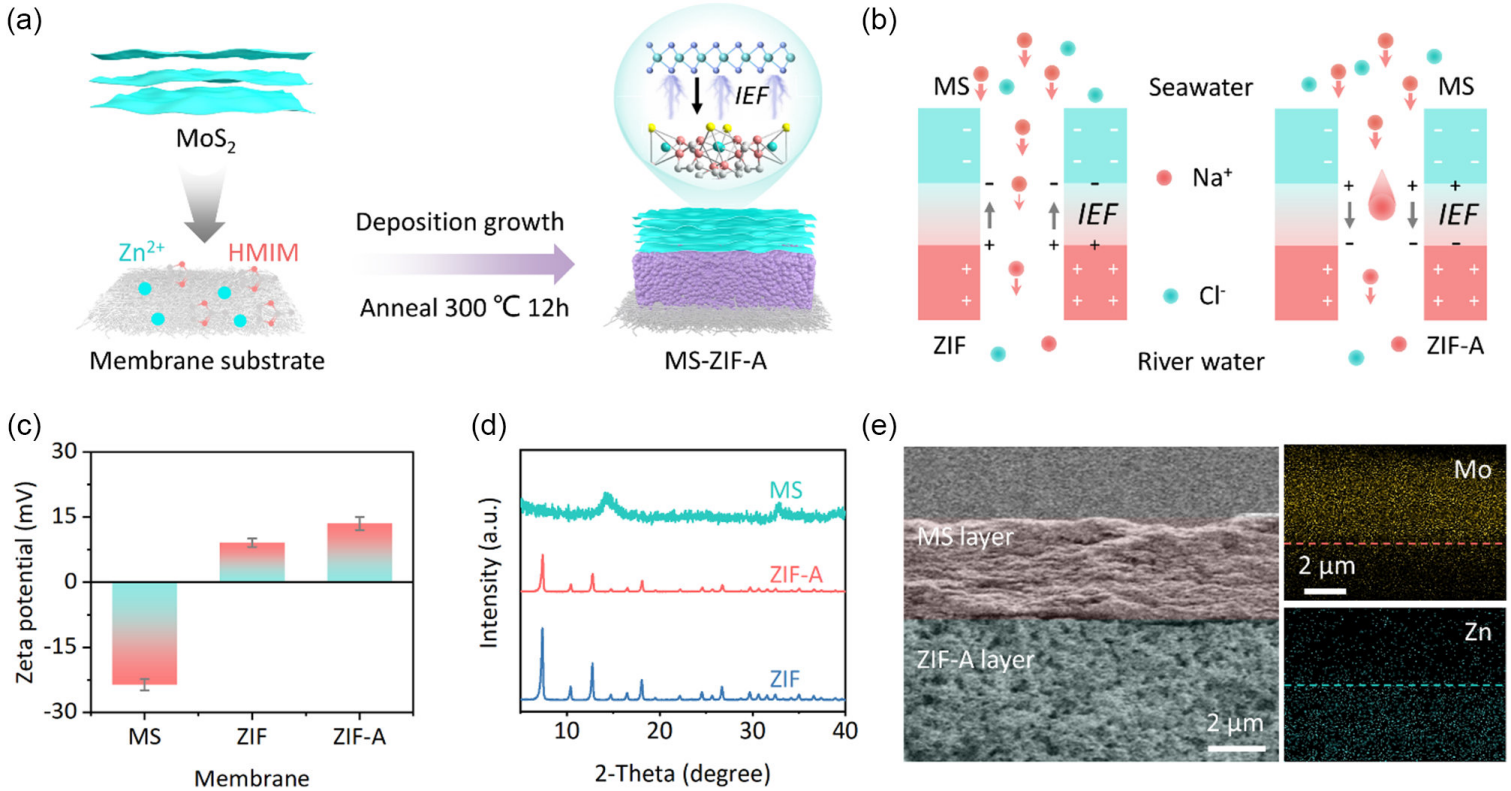
Design, fabrication and structural characterization of the asymmetric MS‐ZIF‐A membrane. (a) Fabrication procedure for the MS‐ZIF‐A asymmetric membrane. (b) Schematic illustration of how the direction of the IEF regulates ion migration in MS‐ZIF and MS‐ZIF‐A membranes. (c) Zeta potentials and (d) XRD patterns of MS, ZIF, and ZIF‐A. Error bars in (c) represent mean ± SD, *n* = 3. (e) Cross‐sectional SEM image of the MS‐ZIF‐A membrane with corresponding EDX elemental maps of Mo and Zn.

The fabrication of the asymmetric MS‐ZIF‐A membrane is schematically shown in Figures 1a and S1. Briefly, a ZIF‐8 layer was first grown on a polydopamine/polyethyleneimine (PDA/PEI)‐functionalized fibrous substrate via a solution‐based growth method. The as‐grown ZIF‐8 layer was then calcined in air at low‐temperature to partially replace coordinated N with O, yielding an oxygen‐doped derivative (denoted ZIF‐A). Subsequently, a negatively charged MoS_2_ thin layer was deposited onto the ZIF‐A substrate by vacuum filtration, producing a bilayer MS‐ZIF‐A membrane with asymmetric charge distribution and pore architecture. The role of the IEF in regulating ionic transport across the asymmetric interface is depicted in Figure 1b. Relative to MS‐ZIF, the IEF in MS‐ZIF‐A is oriented along the ion transport direction, thereby enhancing ion rectification and improving ionic selectivity.

Zeta‐potential measurements confirm that ZIF‐8 (or ZIF‐A) and MoS_2_ exhibit opposite surface‐charge characteristics (Figure 1c), consistent with the intended electrostatic asymmetry in the resulting membrane. Scanning electron microscopy (SEM) images revealed that ZIF‐8 largely retained its granular morphology after low‐temperature calcination (Figure S2). The integrity of the microporous channels after calcination was confirmed through BET analysis (Figure S3). Powder X‐ray diffraction (XRD) patterns collected from the two sides of the membrane further verify the bilayer structure: the MS side exhibits characteristic reflections consistent with MoS_2_, while the ZIF‐A side matches well with simulated ZIF‐8 diffraction features (Figure 1d). Although ZIF‐A and ZIF‐8 share similar peak positions, the reduced peak intensity of ZIF‐A may indicate decreased crystallinity or a lower amount of crystalline ZIF material after calcination, in agreement with prior reports [32]. A cross‐sectional SEM image reveals the MoS_2_ layer and ZIF‐A layer thickness of 3.3 and 4.9 μm, respectively (Figure 1e). Energy‐dispersive X‐ray spectroscopy (EDS) mapping confirms the spatially uniform distribution of Mo and Zn across the membrane, substantiating successful assembly of the asymmetric MS‐ZIF‐A architecture.

**FIGURE 2:**
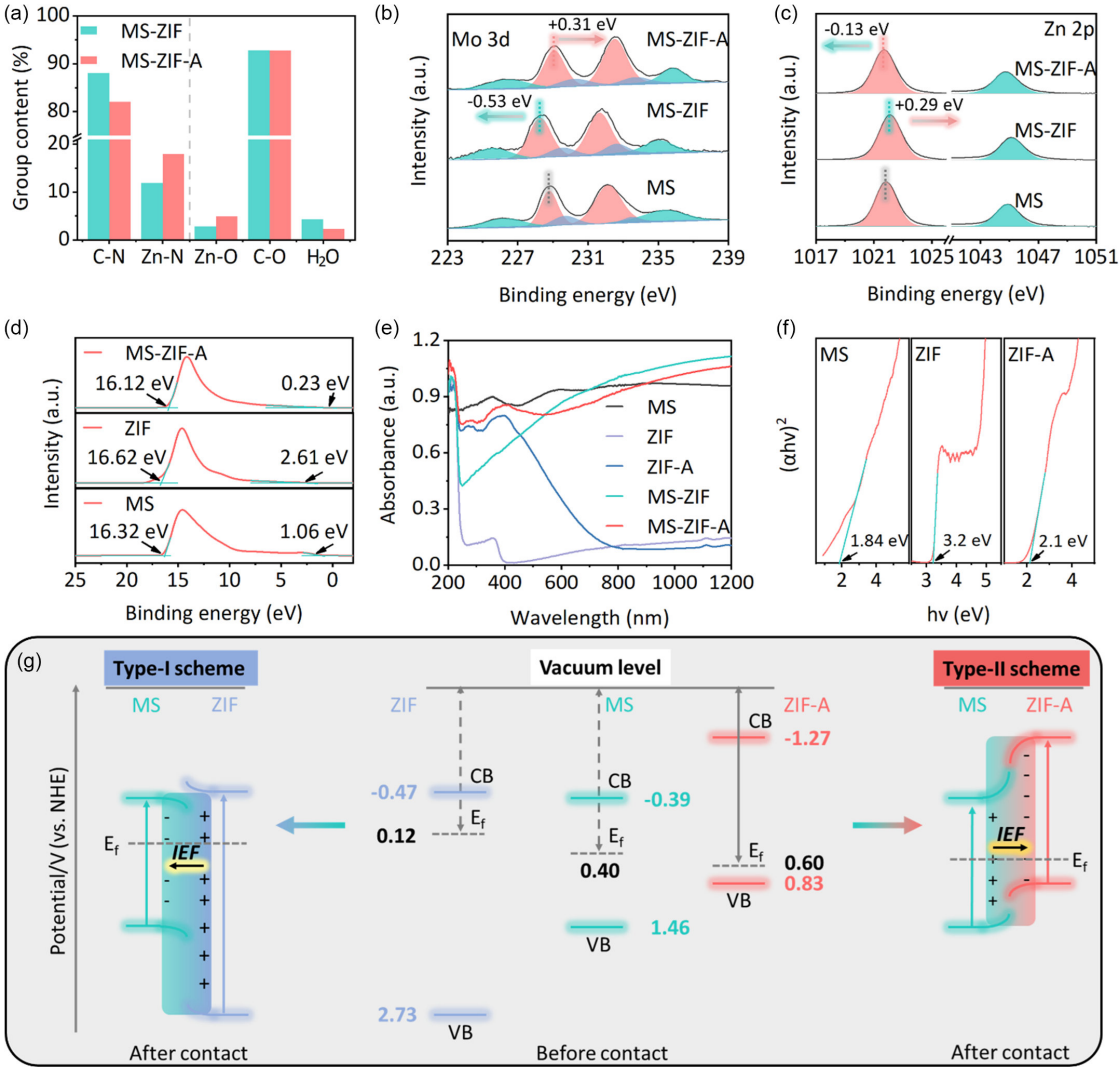
Oxygen‐doping‐modulated electronic structure and interfacial built‐in electric field (IEF). (a) Changes in functional groups of MS‐ZIF and MS‐ZIF‐A based on high‐resolution N 1s and O 1s XPS spectra. High‐resolution XPS spectra of (b) Mo 3d and (c) Zn 2p for MS, MS‐ZIF, and MS‐ZIF‐A. (d) UPS spectra of MS, ZIF, and ZIF‐A used to determine the work function. (e) UV–vis diffuse reflectance spectra and (f) corresponding Tauc plots of MS, ZIF, and ZIF‐A. (g) Schematic comparison of interfacial ionic transfer directions and the resulting IEF in MS‐ZIF and MS‐ZIF‐A heterojunction.

To probe interfacial chemical states and charge redistribution, X‐ray photoelectron spectroscopy (XPS) was performed for MS‐ZIF and MS‐ZIF‐A. In the high‐resolution spectra, the N 1s component at 400.8 eV is assigned to Zn–N coordination, while the O 1s peak at 529.9 eV corresponds to Zn–O bonding (Figure S4). Relative to MS‐ZIF, MS‐ZIF‐A shows a diminished Zn–N contribution accompanied by an enhanced Zn–O signal, consistent with oxygen incorporation during calcination in air at 300 °C and partial formation of Zn–O coordination environments (Figure 2a) [32]. Notably, the Mo 3d and Zn 2p binding energies in MS‐ZIF‐A shift by +0.31 and −0.13 eV, respectively, relative to the corresponding references (Figure 2b,c). In contrast, MS‐ZIF shows the opposite trend (Mo *3d* = −0.53 eV and Zn *2p* = +0.29 eV). Because lower binding energy generally indicates increased local electron density, these opposite shifts indicate that the direction of interfacial electron transfer differs between MS‐ZIF and MS‐ZIF‐A [33]. Specifically, in MS‐ZIF‐A, electron density increases on the ZIF‐A side while decreasing on the MoS_2_ side; in MS‐ZIF, the charge redistribution is reversed. These observations directly support that oxygen doping alters the interfacial electronic interaction and reverses the electron‐transfer direction at the heterojunction [34].

Ultraviolet photoelectron spectroscopy (UPS) was used to quantify the work functions and band structures of MS, ZIF, and ZIF‐A (Figure 2d). The Φ of MS, ZIF, and ZIF‐A were calculated to be 4.90, 4.60, and 5.12 eV, respectively [35]. The increase after oxygen doping is consistent with the higher electronegativity of oxygen (3.44) compared with nitrogen (3.04), which reduces electron density around the Zn coordination environment and decreases the electron‐donating ability of the surface, thereby elevating the work function. The valence band maxima (*E*_VBM_) were calculated to be 5.92, 7.21, and 5.33 eV versus vacuum for MS, ZIF, and ZIF‐A, respectively (corresponding to 1.52, 2.77, and 0.89 V versus the standard hydrogen electrode, SHE) [36]. Ultraviolet–visible diffuse reflectance spectra (UV–vis DRS) show a clear redshift of the absorption edge for ZIF‐A relative to ZIF (Figure 2e), consistent with modified electronic structure upon oxygen incorporation. The optical bandgaps (*E*_g_), extracted using the Kubelka–Munk relationship, were determined to be 1.84, 3.2, and 2.1 eV for MS, ZIF, and ZIF‐A, respectively (Figure 2f) [37]. The corresponding conduction band minima (*E*_CBM_) were calculated to be −0.39, −0.47, and −1.27 V versus NHE for MS, ZIF, and ZIF‐A, respectively. Mott–Schottky (MS) plots of MS, ZIF, and ZIF‐A were also measured to further confirm the energy level positions. As shown in Figure S5, the x‐intercept corresponded to the flat‐band potential (*E*_fb_) of MS, ZIF, and ZIF‐A, which were determined to be –0.39, –0.49, and –1.22 V versus NHE, respectively. Consequently, the *E*_fb_ values of MS, ZIF, and ZIF‐A were found to be close to their corresponding *E*_CBM_ positions. Based on these electronic parameters, the interfacial IEFs in MS‐ZIF and MS‐ZIF‐A were constructed schematically (Figure 2g). For MS‐ZIF‐A, the higher work function of ZIF‐A (5.12 eV) relative to MoS_2_ (4.90 eV) drives electron transfer from MS to ZIF‐A upon contact, producing interfacial charge separation and a IEF oriented toward the ZIF‐A side. In contrast, because ZIF‐8 has a lower work function (4.60 eV) than MoS_2_ (4.90 eV), the MS‐ZIF junction drives electron transfer from ZIF to MS, leading to an oppositely oriented IEF directed toward the MS side. This reversal in IEF direction provides a mechanistic basis for the distinct rectification behavior and ion‐transport performance observed for MS‐ZIF‐A versus MS‐ZIF in subsequent measurements.

The role of the interfacial IEF in modulating ion transport can be understood as follows. In the MS‐ZIF‐A membrane, the IEF is oriented from the MoS_2_ layer to the ZIF‐A layer (Figures 1b and 2g). Under a salinity gradient, the high‐concentration NaCl solution is placed on the MS side. The negatively charged MS surface selectively transports cations (Na^+^) while rejecting anions. As these cations diffuse from MS toward ZIF‐A, they encounter the IEF pointing in the same direction as their diffusion (from MS to ZIF‐A). This IEF exerts an additional electrostatic driving force on cations, lowering their migration resistance across the heterojunction interface. Concurrently, the IEF‐induced band bending facilitates the accumulation of cations at the interface on the ZIF‐A side, while depleting anions in the same region due to the opposite electrostatic force (Figure S6). Once cations enter the positively charged ZIF‐A layer, they are further accumulated due to electrostatic attraction, a phenomenon that, together with the IEF, produces the observed diode‐like rectification. This accumulation–depletion effect enhances the net cation flux and suppresses anion backflow, thereby improving cation selectivity and rectification ratio [38]. In contrast, for MS‐ZIF where the IEF opposes the cation diffusion direction, cations experience an extra energy barrier at the interface, increasing migration resistance and reducing both ion accumulation and selectivity. Thus, the alignment between IEF orientation and ion diffusion direction is the key to achieving enhanced cation transport and higher osmotic energy conversion efficiency.

### 2.2 Salinity Gradient Energy Harvesting Performance

**FIGURE 3:**
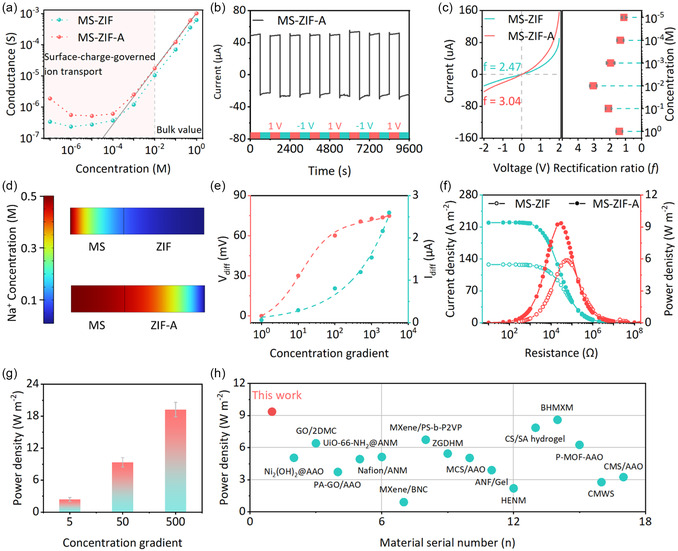
Ion transport rectification and osmotic energy conversion performance. (a) Ionic conductance of MS‐ZIF and MS‐ZIF‐A membrane. (b) *I–T* response of the MS‐ZIF‐A membrane in 0.1 M KCl under an alternating external bias of +1 and −1 V. (c) *I–V* characteristics of MS‐ZIF and MS‐ZIF‐A measured in 10^−4^ M KCl (left), and rectification ratios of MS–ZIF‐A as a function of KCl concentration (right). (d) Simulated ionic concentration profiles for MS‐ZIF and MS‐ZIF‐A under a NaCl gradient. (e) *V*_diff_ and *I*_diff_ recorded under a series of NaCl concentration gradients. (f) Osmotic power output of MS‐ZIF and MS‐ZIF‐A under a 50‐fold NaCl gradient as a function of external load resistance. (g) Output power density of MS‐ZIF‐A under 5‐, 50‐, and 500‐fold NaCl gradients. Error bars represent mean ± SD, *n* = 3. (h) Benchmarking of the peak power density of MS‐ZIF‐A against reported state‐of‐the‐art heterogeneous membranes (see Table S3 for details).

MS‐ZIF and MS‐ZIF‐A were positioned between two electrochemical cells to evaluate their transmembrane ionic transport characteristics by measuring the transmembrane ionic conductivity at various concentrations. KCl was selected as the probe electrolyte due to the similar ionic mobility and volume of K^+^ and Cl^−^. The MS‐ZIF‐A membrane exhibits higher conductivity compared to the MS‐ZIF membrane (Figure 3a). As KCl concentration decreased, the ionic conductivity of the MS‐ZIF‐A membrane deviated from the bulk value (black dashed line) at concentrations below 0.1 M, indicating that surface charge controls ion transport at low concentrations. Notably, at concentrations below 10^−5^ M, the overlap of the electric double layer led to an increase in conductivity. To verify cation selectivity, 1 M and 10^−6^ M KCl solutions were applied to the MS and ZIF‐A sides, respectively. Given the substantial concentration difference between the two solutions, ionic current was predominantly attributed to K^+^ or Cl^−^ transfer from the MS side to the ZIF‐A side. When the anode was placed on the MS side, the forward current direction aligned with the concentration gradient, with K^+^ dominating the ionic current. Conversely, when the anode was placed on the ZIF‐A side, the forward current direction opposed the concentration gradient, with Cl^−^ dominating the ionic flow [39]. As shown in Figure S7, the K^+^ dominated ionic current was significantly greater than the Cl^−^ dominated current, confirming the cation selectivity of the MS‐ZIF‐A membrane. To further confirm the cation selectivity of MS‐ZIF‐A, current–time (*I*–*T*) tests were performed by alternating +1 and −1 V external biases. As shown in Figure 3b, after switching between positive and negative biases, K^+^ (forward current) exhibited higher current than Cl^−^ (reverse current), with stable current retention, demonstrating the excellent cation transport stability of the MS‐ZIF‐A membrane. We further investigated the ICR behavior of the MS‐ZIF‐A membrane at different KCl concentrations (Figure 3c, left). As the KCl concentration increased, the rectification ratio initially increased and then decreased due to the surface charge effects at the hybrid membrane–electrolyte interface (Figure 3c, right) [40]. When the KCl concentration reached 10^−2^ M, the rectification ratio of the MS‐ZIF‐A membrane peaked at 3.04, showing superior rectification performance compared to MS‐ZIF.

When the ion‐selective membrane is applied to a transmembrane salinity gradient system (Figure S8), the salt concentration difference across the membrane continuously drives ions from the high‐concentration side to the low‐concentration side. Due to the high cation selectivity and the forward‐driven IEF of the membrane, cations preferentially pass through and accumulate on the low‐concentration side, while anions accumulate on the high‐concentration side, generating a transmembrane potential difference. COMSOL simulations of NaCl transport through MS‐ZIF and MS‐ZIF‐A membranes were performed under asymmetric concentration conditions (0.5 M NaCl at the MS side vs. 0.01 M at the opposite side) (Figures S9 and S10). For MS‐ZIF, the reverse‐driven IEF hinders the transmembrane transport of both cations and anions, reducing the conversion of Gibbs free energy into electrical energy (Figure 3d). In contrast, MS‐ZIF‐A reveals substantially enhanced interfacial ion accumulation relative to MS‐ZIF due to enhanced IEF. When applying a transmembrane salinity gradient system to an asymmetric ion‐selective membrane, the open‐circuit voltage (*V*_oc_) and short‐circuit current (*I*_sc_) can be observed on the measured *I–V* curve axes, and the internal resistance is determined using *R*_channel_ = *V*_oc_/*I*_sc_. When the high‐concentration solution was on the MS side, the *I–V* curve of MS‐ZIF‐A under a 0.5/0.01 M NaCl gradient showed an *I*_sc_ of 6.2 μA, a *V*_oc_ of 176 mV, and an *R*_channel_ ≈ 28 kΩ. These values were superior to those of MS‐ZIF (*I*_sc_ = 3.6 μA, *V*_oc_ = 153 mV, *R*_channel_ ≈ 43 kΩ), suggesting that the interfacial electric field in MS‐ZIF‐A facilitates directional ion transport (Figure S11, solid lines). In contrast, under the reverse concentration gradients, both *I*_sc_ and *V*_oc_ decreased substantially, while the ionic transport resistance increased to approximately 35 and 51 kΩ for MS‐ZIF and MS‐ZIF‐A, respectively (Figure S11, dashed lines). This further confirms the polarity of the membrane structure and the preferred ion diffusion direction. To elucidate how tuning the work function of the functional layer affects ion diffusion, we compared the *V*_oc_ and *I*_sc_ of asymmetric membranes calcined at different temperatures (Figure S12). As the temperature increased from 200 to 300 °C, the energy band alignment between MS and ZIF gradually improved, with *V*_oc_ and *I*_sc_ increasing from 126 mV and 4.1 μA to 175 mV and 6.2 μA, respectively. This result further indicates the enhancement of IEF in ion transport. Electrochemical impedance spectroscopy (EIS) was performed to investigate the charge‐transfer resistance of the asymmetric MS‐ZIF and MS‐ZIF‐A membranes (Figure S13). The Nyquist plots for MS‐ZIF and MS‐ZIF‐A show corresponding semicircles and sloped lines in the high and low‐frequency regions. Compared to MS‐ZIF, the MS‐ZIF‐A membrane exhibited lower ionic resistance. The net diffusion current (*I*_diff_) and diffusion potential (*E*_diff_) were corrected by subtracting the contribution from the electrode redox potential (*E*_redox_) (Figure S14). As shown in Figure 3e and Table S1, both *V*_diff_ and *I*_diff_ values increased with the concentration gradient. Table S2 calculates the cation transfer number (*t*_*+*_) and energy conversion efficiency (*η*) for MS‐ZIF‐A at corresponding concentrations. At a concentration gradient of 10: 1, the *t*_*+*_ was 0.90, with a maximum *η* of 32.3%. As the concentration gradient increased, both the *t*_*+*_ (0.77) and *η* (15.1%) significantly decreased, which can be attributed to the shielding of surface charge at higher concentration gradients.

To quantify the energy harvested by the asymmetric membrane under a salinity gradient, the generated electrical energy was delivered to an external circuit through a variable load resistance (*R*_*L*_). The areal power output was calculated as *P* = *I* ^2^ × *R*_L_, where *I* is the measured current at a given salinity gradient. As *R*_L_ increased, *I* decreased, and the power density exhibited a maximum when *R*_L_ matched the internal resistance. Under a 50‐fold NaCl gradient, the optimized MS‐ZIF‐A membrane reached a peak power density of ~9.4 W m^−2^ (Figure 3f), substantially higher than that of MS‐ZIF (5.9 W m^−2^). Furthermore, different membranes were prepared by adjusting the loading amount of MS on ZIF‐A to vary (Figure S15). The MS‐ZIF‐A membrane exhibited the highest output power density and the lowest transport resistance. To assess energy extraction from representative natural waters, we further evaluated MS‐ZIF‐A under salinity gradients of 5 and 500. Maximum power densities of 2.4 and 19.2 W m^−2^ were obtained (Figures 3g and S16), highlighting the broad applicability of MS‐ZIF‐A across realistic salinity ranges. Notably, MS‐ZIF‐A outperformed most previously reported asymmetric membranes operated at comparable concentration gradients (Figure 3h and Table S3).

### 2.3 Photocoupled Energy Harvesting and Wastewater Remediation

**FIGURE 4:**
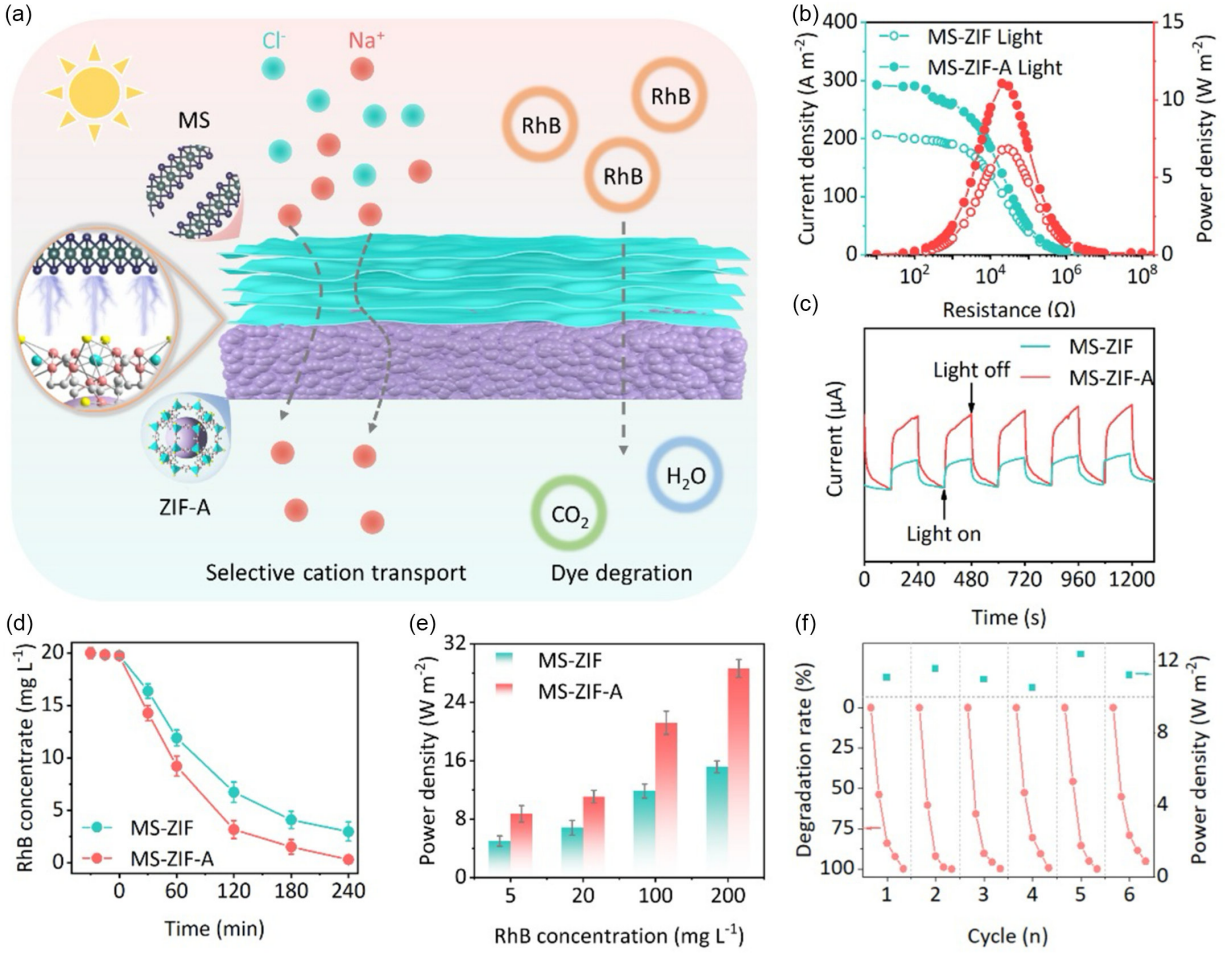
Photocoupled salinity gradient energy conversion with simultaneous dye degradation. (a) Schematic of the photocoupled system integrating RhB photodegradation and salinity gradient power generation using the MS‐ZIF‐A membrane. (b) Osmotic power output of MS‐ZIF and MS‐ZIF‐A in a saline RhB effluent/riverwater configuration under light irradiation. (c) *I*–*T* response of MS‐ZIF‐A during on/off light cycling. (d) Time‐dependent RhB degradation over MS‐ZIF and MS‐ZIF‐A under illumination. Error bars represent mean ± SD, *n* = 3. (e) Output power density of MS‐ZIF and MS‐ZIF‐A as a function of RhB concentration. Error bars represent mean ± SD, *n* = 3. (f) Cycling stability of MS‐ZIF‐A, showing RhB degradation efficiency and output power density over repeated operation.

MoS_2_ is a widely explored photocatalyst for environmental remediation, including the degradation of organic pollutant. As shown in Figure 2e, the UV–vis DRS of MS‐ZIF‐A display a pronounced redshift of the absorption edge relative to MS‐ZIF, indicating enhanced light harvesting that is expected to promote photocatalytic activity. This synergy between the MS and ZIF‐A layers enables a coupled strategy in which polluted, high‐salinity wastewater serves as the concentrated feed, allowing simultaneous electricity generation and pollutant degradation. We therefore investigated the use of these asymmetric membranes for photocatalytic treatment of saline dye wastewater while harvesting osmotic energy. As illustrated in Figure 4a, the MS side was contacted with 20 mg L^−1^ RhB solution containing 0.5 M NaCl, whereas the ZIF‐A side faced 0.01 M NaCl solution. Prior to illumination, the membranes were equilibrated in the dark to establish adsorption–‍‍desorption equilibrium. Under irradiation, MS‐ZIF‐A delivered a peak power density of 11.1 W m^−2^ (Figure 4b). To examine light responsiveness, we recorded *I–T* traces under chopped illumination (Figure 4c). Increasing the light intensity enhanced the power density from 9.4 to 15.1 W m^−2^ (Figure S17). Under simulated sunlight, the current increased immediately without an observable delay, and the photocurrent switching rate of MS‐ZIF‐A was 1.22 times that of MS‐ZIF, indicating more efficient photoassisted ion transport. We attribute this improvement to the interfacial band alignment in MS‐ZIF‐A, which promotes the separation and transfer of photogenerated carriers and thereby facilitates cation migration across the membrane under illumination. In addition, interfacial electron transfer to MoS_2_ can increase its surface charge density under light, consistent with prior reports, thereby strengthening ion selectivity and ion flux [41, 42]. By contrast, the less favorable band alignment in MS‐ZIF yields a larger Schottky barrier, which suppresses carrier separation and limits photoinduced enhancement. The increased surface charge density under illumination can also strengthen electrostatic interactions with cationic RhB, further accelerating its degradation.

As shown in Figure 4d, MS‐ZIF‐A exhibited superior performance to MS‐ZIF. To elucidate the mechsnism of pollutant degradation and osmotic energy conversion during the photocoupled process, a series of control experiments (dark adsorption, membrane‐free, single‐component, and salinity gradient‐free) were performed (Figures S18–S20). The excellent degradation and osmotic energy harvesting performance of MS‐ZIF‐A was revealed to originate from the combined effects of favorable IEF orientation, modified electronic structure, and enhanced photoinduced charge separation. RhB removal is mainly governed by the photocatalytic activity of MoS_2_, which can promote reactive oxygen species generation (•O_2_^−^ and ·OH) [43]. LC‐MS analysis indicated the formation of low‐molecule‐weight intermediates, while the TOC removal efficiency of 52.3% confirmed the partial mineralization into CO_2_ and H_2_O of RhB (Figures S21−S23). Because industrial saline wastewaters commonly contain 0.4–1.5 M salts, we next assessed performance in RhB solutions with NaCl concentrations of 0.4, 0.9, and 1.5 M. The power density of MS‐ZIF‐A increased from 11.1 to 16.6 W m^−2^ as salt concentration rose (Figure S24). Increasing RhB from 5 to 200 mg L^−1^ further raised the maximum power density to 30 W m^−2^ (Figure 4e), which we attribute to the increased osmotic pressure at higher solute concentrations (van't Hoff equation), thereby strengthening the driving force for ion transport and interfacial reactions. Notably, introducing organic pollutants increased the power output, even though photocatalytic degradation consumes a fraction of the photogenerated carriers. Finally, we assessed membrane stability under optimized conditions. After six consecutive cycles, MS‐ZIF‐A maintained efficient RhB degradation and stable power output (Figure 4f), underscoring its promise for energy‐positive treatment of saline industrial wastewater while simultaneously harvesting salinity gradient energy. As shown in Figure S25, the permeation energy collection performance remained remarkably stable throughout the 36 h operation period, with no obvious degradation. Furthermore, the original morphological structure and chemical composition MS‐ZIF‐A remained unchanged after continuous operation of MS‐ZIF‐A for 36 h. The concentrations of dissolved Mo and Ni in the solution were determined to be 5.4 and 12.5 μg L^−1^, respectively (Figure S26). Both values were substantially lower than the corresponding regulatory limits (e.g., 0.5 mg L^−1^ for Mo under the GB 8978‐1996 Class I standard; 1.5 mg L^−1^ for Zn under GB 21 900‐2008 for existing enterprises). This confirmed that the MS‐ZIF‐A membrane exhibited outstanding structural stability.

## 3 Conclusion

In conclusion, we developed an asymmetric MS‐ZIF‐A membrane that integrates a IEF to accelerate selective ion transport and thereby improve osmotic energy conversion. By matching the work function of the functional layers, the interfacial band alignment strengthens the internal driving force for ion migration, enabling a peak power density of 9.4 W m^−2^ under a 50‐fold NaCl salinity gradient. Beyond energy harvesting from salinity differences, the membrane also operates in simulated industrial wastewater containing metal ions and organic contaminants, delivering power densities up to 30 W m^−2^ while simultaneously promoting pollutant degradation. These results highlight work function engineering as an effective strategy to construct interfacial IEF in asymmetric membranes, providing a general design principle for high‐performance blue‐energy devices and offering a promising route toward energy‐positive treatment of saline wastewater.

## Funding

This study was supported by Natural Science Foundation of China (52300201), ARC DECRA project (DE22001577), JST‐ERATO project (JPMJER2003), and ARC Laureate Fellowship (FL230100095). This work used the Queensland node of the NCRIS‐enabled Australian National Fabrication Facility (ANFF).

## Conflicts of Interest

The authors declare no conflicts of interest.

## Supporting information

Supplementary Material

## Data Availability

The data that support the findings of this study are available from the corresponding author upon reasonable request.
