# Supplementary material for: Engineering Interfacial Built‐In Electric Fields via Work Function Matching Enables Photocoupled Osmotic Energy Recovery From Saline Dye Wastewater
Source: Small Sci. 2026 Jul 23;6(7):e70345. doi: 10.1002/smsc.70345 (PMC13410391; doi:10.1002/smsc.70345)
Supplement: Supplementary file 1 — Supplementary Material [file SMSC-6-e70345-s001.pdf]

Engineering interfacial built-in electric fields via work-function matching enables photocoupled osmotic energy recovery from saline dye wastewater

Weixiang Tao[a], Peifang Wang[a], Gang Zhou[a], Yanhui Ao[a], Jie Wang[a,*], Yusuke Yamauchi[b,c,d,*]

a. Key Laboratory of Integrated Regulation and Resource Development on Shallow Lakes, Ministry of Education, College of Environment, Hohai University, Nanjing 210098, China.

b. Australian Institute for Bioengineering and Nanotechnology (AIBN), The University of Queensland, Brisbane, Queensland 4072, Australia.

c. Department of Materials Process Engineering, Graduate School of Engineering, Nagoya University, Nagoya 464-8603, Japan

d. Department of Convergent Biotechnology and Advanced Materials Science, Kyung Hee University, 1732 Deogyeong-daero, Giheung-gu, Yongin-si, Gyeonggi-do, 17104, South Korea

E-mail: wang.jie@hhu.edu.cn (J. W.); y.yamauchi@uq.edu.au (Y. Y.)

Methods

1. Reagents and materials

Zinc chloride ($ZnCl_2$), Methanol (MeOH), sodium formate (HCOONa), potassium chloride (KCl), sodium chloride (NaCl), and Rhodamine B (RhB) were purchased from Sinopharm Chemical Reagent Co., Ltd., Tris-hydrochloride (Tris-HCl) buffer, dopamine hydrochloride, polyethyleneimine (PEI) and 2-methylimidazole (HMIM) were obtained from Aladdin. Quartz fiber membrane were supplied by Membrane Space. A MoS_2 nanosheet dispersion (1 mg mL^{-1}, XF141) was purchased from Jiangsu Xianfeng Nanomaterials Technology Co., Ltd. Deionized water (18 MΩ · cm) was used throughout. All chemicals were used as received without further purification.

2. Synthesis of MS-ZIF-A membrane

Quartz fiber membrane were pretreated with dilute HCl, rinsed thoroughly with deionized water, and then immersed in tris-HCl buffer (0.05 M, pH 8.5) containing dopamine hydrochloride (2 mg mL^{-1}) and PEI (2 mg mL^{-1}). The mixture was shaken at room temperature for 4 h to form an adhesive polydopamine/PEI interlayer. The membranes were then transferred into a methanolic HMIM solution (5 M) and incubated at 50 °C for 2 h to generate nucleation sites for ZIF growth.

ZIF-8 was grown following a reported procedure [1]. Briefly, $ZnCl_2$ (2.152 g), HMIM (2.592 g), and HCOONa (1.072 g) were dissolved in methanol (80 mL) under stirring. The pretreated membranes were placed horizontally in a Teflon-lined autoclave containing the precursor solution and reacted at 85 °C for 6 h. After natural cooling to room temperature, the membranes were rinsed with methanol and dried at 60 °C. To obtain oxygen-doped ZIF-8 (denoted ZIF-A), the dried ZIF-8 membranes were calcined in air by heating to 300 °C at 5 °C min^{-1} and holding for 12 h [2]. Subsequently, MoS_2 nanosheets were deposited onto the ZIF-A side by vacuum filtration (20 mL of 1 mg mL^{-1} dispersion). The resulting membranes were annealed at 90 °C (5 °C min^{-1}, 2 h) in a tube furnace to improve interfacial contact, yielding MS-ZIF-A membranes. For comparison, MS-ZIF membranes were prepared by depositing MoS_2 nanosheets onto uncalcined ZIF-8 membranes using the same vacuum-filtration.

3. Characterization

Membrane morphology was examined by field-emission scanning electron microscopy (FESEM, Hitachi S-4800). X-ray photoelectron spectroscopy (XPS) and ultraviolet photoelectron spectroscopy

(UPS) were performed on a Thermo Scientific ESCALAB 250 Xi using an Al Kα X-ray source (hν = 1486.6 eV) and a hemispherical analyzer. Binding energies were calibrated to the C *1s* peak at 284.8 eV, and peak fitting was conducted using Avantage software. Surface potential distributions were measured by Kelvin probe force microscopy (KPFM) in atomic force microscopy mode (Bruker Dimension Icon). X-ray diffraction (XRD) patterns were collected on a Bruker D8 Focus diffractometer with Cu Kα radiation (λ = 1.5406 Å) and a Lynx-Eye detector. Zeta potential as a function of pH were measured using a Malvern ZS90 instrument. The concentration of Mo and Zn in the solution was analyzed by inductive coupled plasma mass spectrometry (ICP-MS, Agilent 7800). BET apparatus (ASAP2460) was employed to measure specific surface area (BET) of the samples. The mineralization rate of RhB was determined using a total organic carbon analyzer (TOC, Xpert TOC TN). Furthermore, the degradation intermediates were analyzed by a LC-MS (Anglient 1260–6210). UV-vis diffuse reflectance spectra (DRS) were recorded on a Bruker ESP 500 spectrometer.

The secondary electron cutoff energy (E_{cutoff}) were determined from the x-axis intercept of the extrapolated cutoff edges, yielding E_{cutoff} values of 16.32, 16.62, and 16.12 eV for MS, ZIF, and ZIF-A, respectively. The work functions were calculated according to Equation (1) (hv = 21.22 eV),

$$\Phi = hv - E_{cutoff} = E_f - E_{vac} \quad (1).$$

The valence band maxima (E_{VBM}) were obtained using Equation (2):

$$E_{VBM} = hv - (E_{cutoff} - E_{onset}) \quad (2).$$

The optical bandgaps (E_g), extracted using the Kubelka-Munk relationship (Equation 3):

$$\alpha hv = A\,(hv - E_g)^2 \quad (3).$$

The corresponding conduction band minima (E_{CBM}) were calculated from Equation (4),

$$E_{CBM} = E_{VBM} - E_g \quad (4).$$

4. Electrical measurements

Ion transport and osmotic energy conversion were evaluated using a Keithley 6487 picoammeter/voltage source. The membrane was sealed between two chambers of a custom electrochemical cell, with an effective membrane area of 3×10^{-8} m^2. Homemade Ag/AgCl electrodes were used as the working and counter electrodes. Current-voltage (*I-V*) curves were recorded by sweeping the voltage from −1 to +1 V with a step of 0.02 V. In symmetric KCl solutions (0.01 mM to 1 M), the ionic conductance was obtained from the slope of the *I-V* curves. Under concentration

gradients, the open-circuit voltage (V_{oc}) and short-circuit current (I_{sc}) were determined from the intercepts of the *I-V* curves. For power-output measurements, the Ag/AgCl electrodes were connected to an external variable load resistor (MC-21-B, Mingcheng Co., Shenzhen, China), and the load current was continuously recorded. In photoresponse measurements, an H-shaped cell was employed. An acrylic disk with a diameter of 2 cm and the thickness of 1 mm was custom-made, featuring a central hole with an area of 3×10^{-8} m^2. Transmembrane ionic currents were recorded using Keithley 6487 electrometer and Ag/AgCl electrodes under varying electrolyte concentrations and in the presence or absence of light. A CMH-250 xenon lamp (Beijing Andit Optoelectronic Technology Co., Ltd.) was used as the light source to simulate solar irradiation. All electrolyte solutions were prepared using ultrapure water with a resistivity of 18.2 MΩ·cm, and measurements were performed at room temperature. RhB was selected as model organic pollutant. The effects of light intensity, salt concentration, and dye concentration on ion transport and osmotic energy output were systematically investigated. Furthermore, various scavengers including p-benzoquinone (p-BQ), ammonium oxalate (AO) and isopropanol (IPA) were employed to identify the dominant reactive oxygen species involved in the photodegradation processes of both RhB. In degradation cycling tests, the membrane was cleaned and remeasured after every 2 h of illumination, for a total of 6 cycles. Additionally, a continuous stability test was conducted under constant external load of 20 kΩ using a 50-fold NaCl concentration gradient (0.5 M/0.01 M), with the high-concentration side containing 20 mg L^{-1} of RhB. Both solutions had a volume of 5 L and were circulated between the two chambers by a peristaltic pump at a flow rate of 5 mL min^{-1} to maintain a stable concentration gradient. The test was run continuously for 36 h.

5. Electrode calibration

Transmembrane *I-V* measurements under concentration gradients were analyzed using an equivalent circuit model. The measured open-circuit voltage (V_{oc}) was separated into two contributions: a redox-related potential (E_{redox}) arising from interfacial inhomogeneity at the electrode-electrolyte interface and a diffusion potential (E_{diff}) originating from membrane ion selectivity. To quantify E_{redox}, a nonselective single-pore silicon membrane was used in place of the ion-selective membrane, and the results are summarized in Table S1.

6. Energy conversion efficiency

Owing to the negatively charged membrane surface, the membrane exhibits preferential transport of cations under a given salinity gradient. The cation transference number (t_+) was estimated from the measured diffusion potential (E_{diff}) based on the Nernst–Planck description:[3]

$$t_+=\frac{1}{2}\left(\frac{E_{diff}}{\frac{RT}{zF}Ln\left(\frac{\gamma_H c_H}{\gamma_L c_L}\right)}+1\right),$$

where E_{diff} is the diffusion potential, R is the universal gas constant, T is the absolute temperature, F is the Faraday constant, z is the ionic charge number, γ is the activity coefficient, and c is the ion concentration. Subscripts H and L denote the high- and low-concentration reservoirs, respectively.

The energy conversion efficiency (η) was defined as the ratio of the electrical energy output to the thermodynamic energy input (the Gibbs free energy of mixing). The maximum theoretical efficiency at the matched-load condition can be expressed as:

$$\eta=\frac{1}{2}(2t_+-1)^2.$$

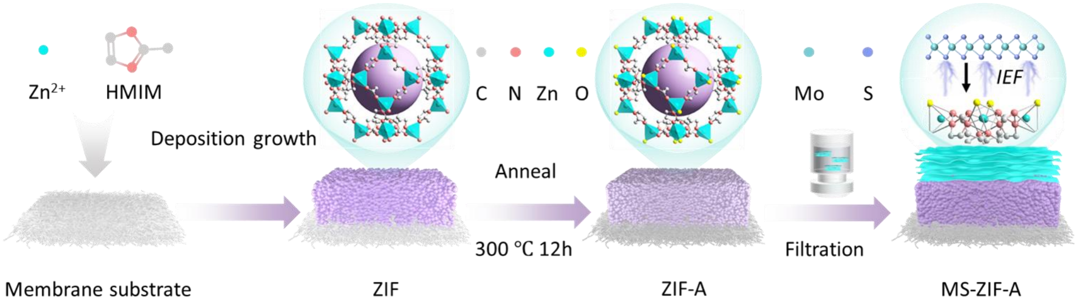

Figure S1. Schematic illustration of the step-by-step fabrication process of the MS-ZIF-A hybrid membrane.

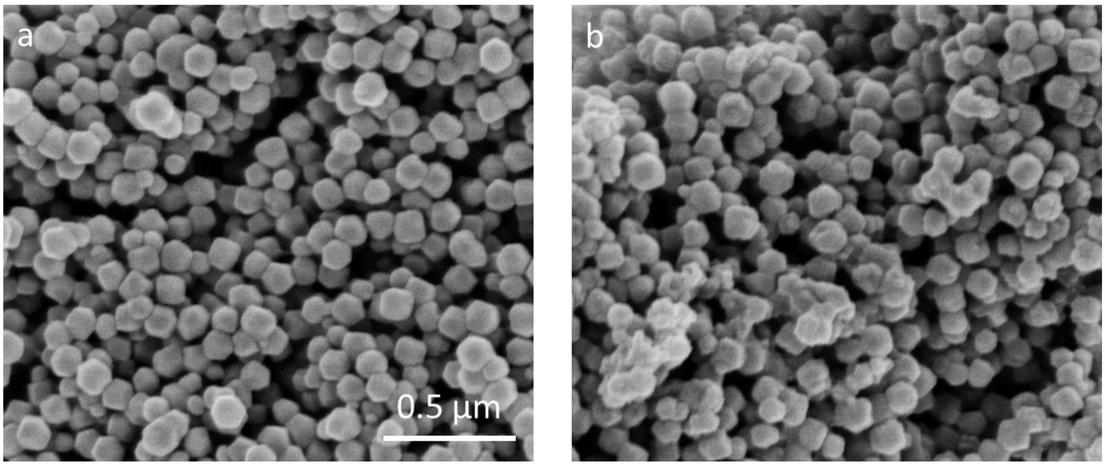

Figure S2. SEM images of (a) ZIF and (b) ZIF-A.

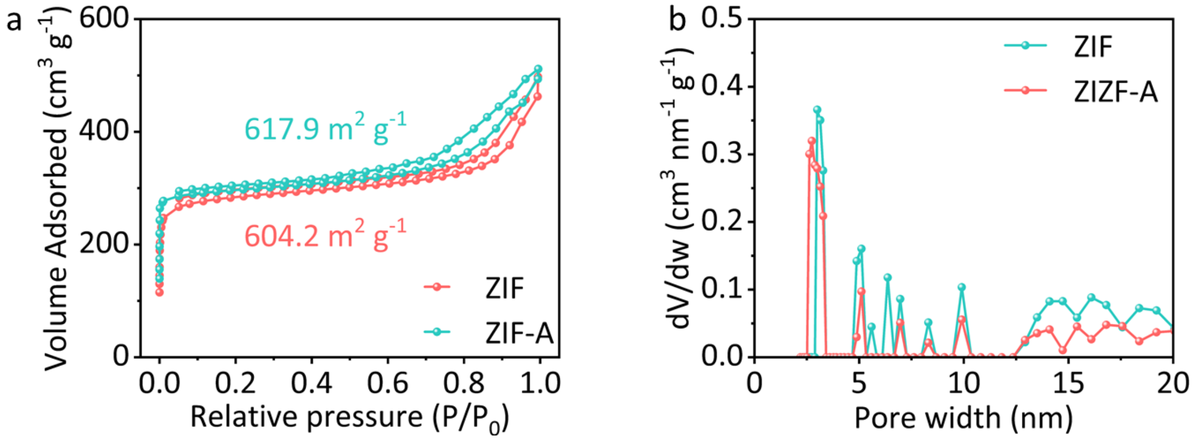

Figure S3. (a) Nitrogen adsorption and desorption isotherms and (b) the corresponding pore size distributions of ZIF and ZIF-A.

Note: The specific surface areas of ZIF and ZIF-A were 617.9 m^2 g^{-1} and 604.2 m^2 g^{-1} respectively, and the pore size distribution remained unchanged after calcination at 300 °C, indicating that the microporous channels remained open and intact.

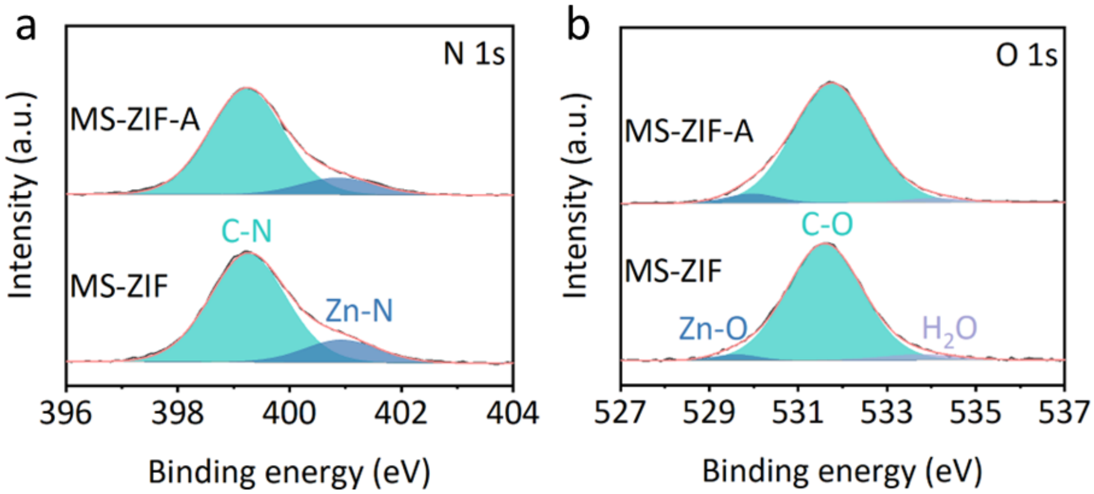

Figure S4. High-resolution (a) N *1s* and (b) O *1s* XPS spectra of MS-ZIF and MS-ZIF-A.

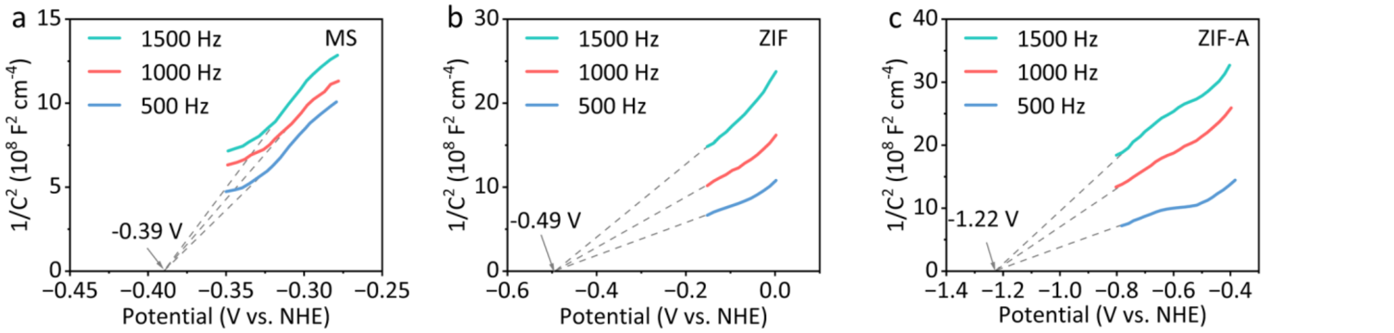

Figure S5. Mott-Schottky plots of (a) MS, (b) ZIF, and (c) ZIF-A tested at different frequencies.

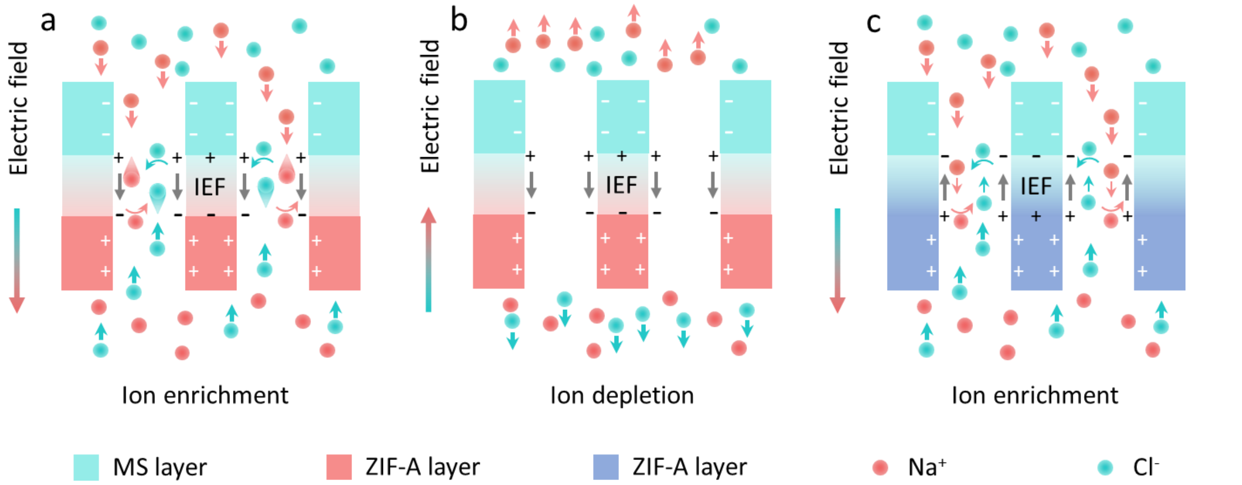

Figure S6. Schematic illustration of ion enrichment and ion depletion that give rise to the ionic diode effect in (a, b) MS-ZIF-A and (c) MS-ZIF membrane.

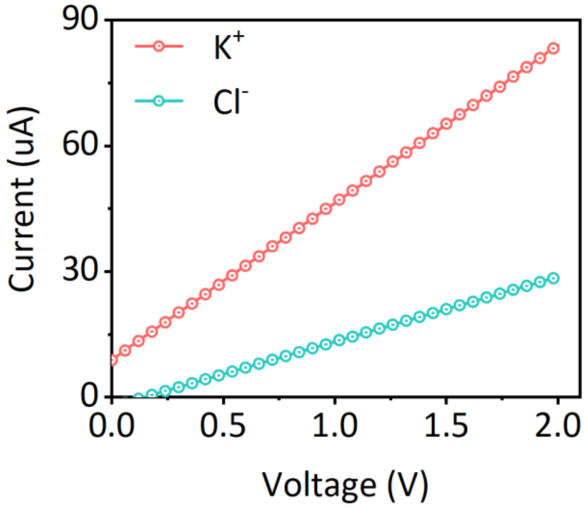

Figure S7. *I*–*V* curves of the MS-ZIF-A membrane measured with 1 M KCl and 1 μM KCl solutions in the two half-cells (MS side and ZIF-A side, respectively).

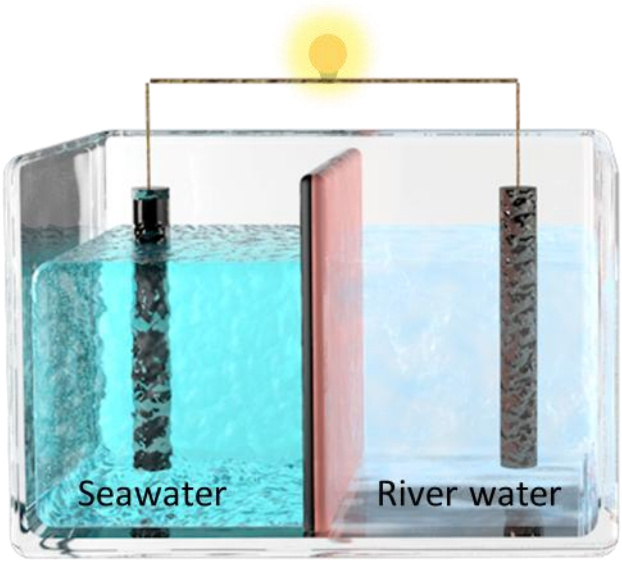

Figure S8. Schematic illustration of the custom electrochemical cell used to evaluate osmotic energy conversion under a salinity gradient.

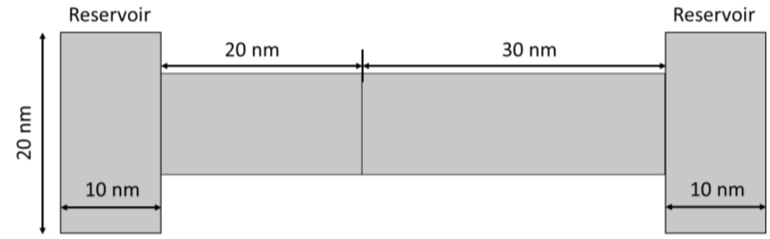

Figure S9. Schematic of the idealized composite nanochannel model used for numerical simulations (not to scale). A cylindrical channel with a length of 20 nm and diameter of 10 nm was used to represent the MS layer, and a second cylindrical channel with a length of 40 nm and the same diameter was used to represent the ZIF (or ZIF-A) layer. Both ends of the nanochannel were connected to electrolyte reservoirs (20 nm × 10 nm) to minimize inlet/outlet mass-transfer resistance.

Numerical simulations were performed using COMSOL Multiphysics 6.2 (COMSOL Inc.).[4-6] In the macroscopic analysis, the electrostatic physics field of the AC/DC module and the transport of diluted species physics field of the chemical species transport module were used. The advantage of employing the COMSOL software in this simulation analysis was that its rich and diverse physics interfaces allowed for easier selection of appropriate physics fields, enabling the simulation and analysis of interactions among multiple physics fields. Consequently, multiphysics coupling was achieved, leading to more accurate simulation results and intuitive visualization of post-processing graphs.

Establishing reasonable assumptions was crucial in performing the simulation analysis, as they simplified the model and made the problem solvable while retaining the key characteristics of the phenomenon under study. The following assumptions were made in this simulation analysis:

(1) Current continuity and charge conservation were maintained within the analysis domain.

(2) Free diffusion and electromigration diffusion were considered as the transport modes for chemical species.

(3) The system was assumed to satisfy the transient assumption.

Geometry creation was a fundamental step in building the simulation model, as it involved defining the physical shape and spatial layout of the model. Based on the complexity of the model in this simulation, a two-dimensional geometry was selected to facilitate subsequent computation and convergence, and it was used for simulation analyses in follow-up studies.

Different diffusion coefficients were obtained for MoS_2, ZIF and ZIF-A due to their structural differences.

The physical phenomena involved in the simulation were directly defined by the physical field interfaces. The accuracy and reliability of the simulation results were ensured by the selection of appropriate interfaces. In this simulation analysis, the electrostatic physics field of the AC/DC module was selected. The zeta potentials of MoS_2, ZIF and ZIF-A were −23.6 mV, 9.18 mV, and 13.6 mV, respectively. Two key equations were employed by Gauss's law for electricity and Coulomb's law.

Gauss's law:

$$\nabla\cdot \mathrm{D} = \rho_v$$

D is the electric displacement vector (C m^{-2}), ρ_v is the charge density (C m^{-3}).

Coulomb's Law:

$$\mathrm{E} = -\nabla \mathrm{V}$$

Here, E represents the electric field strength (V m^{-1}), v represents the electric potential (V). It indicates that the electric field strength E is the negative value of the electric potential gradient.

In the electrochemical system, the influence of potential variations on species migration was considered. Consequently, the Diluted Species transport physics interface within the Chemical Species Transport module was selected. The high concentration side (0.5 M) was located at the molybdenum sulfide side, whereas the low concentration side was set to 0.01 M. A potential coupling interface was then added to couple the electrostatic and diluted species transport physics. The Nernst-Planck equation, which combines Fick's law of diffusion and ionic migration, was employed to describe mass transport in the electrochemical system.

The conservation equation of matter:

$$\nabla\cdot \mathrm{J} = R$$

J is the diffusion flux (mol $(m^2\cdot s)^{-1}$), R is the source term (mol $(m^3\cdot s)^{-1}$), representing the rate of mole generation or consumption due to chemical reactions or sources or sinks within a unit volume.

$$\mathrm{J} = -D\nabla c - z\mu F c\nabla V$$

Here, J represents the flux of the substance (mol/(m2·s)), D represents the diffusion coefficient of the substance (m^2 s^{-1}), c represents the concentration of the substance (mol m^{-3}), z represents the charge of ionic substances, u represents the mobility of charged substances (m^2 $(V\cdot s)^{-1}$), F represents the Faraday constant (C mol^{-1}), and V represents the electric potential (V).

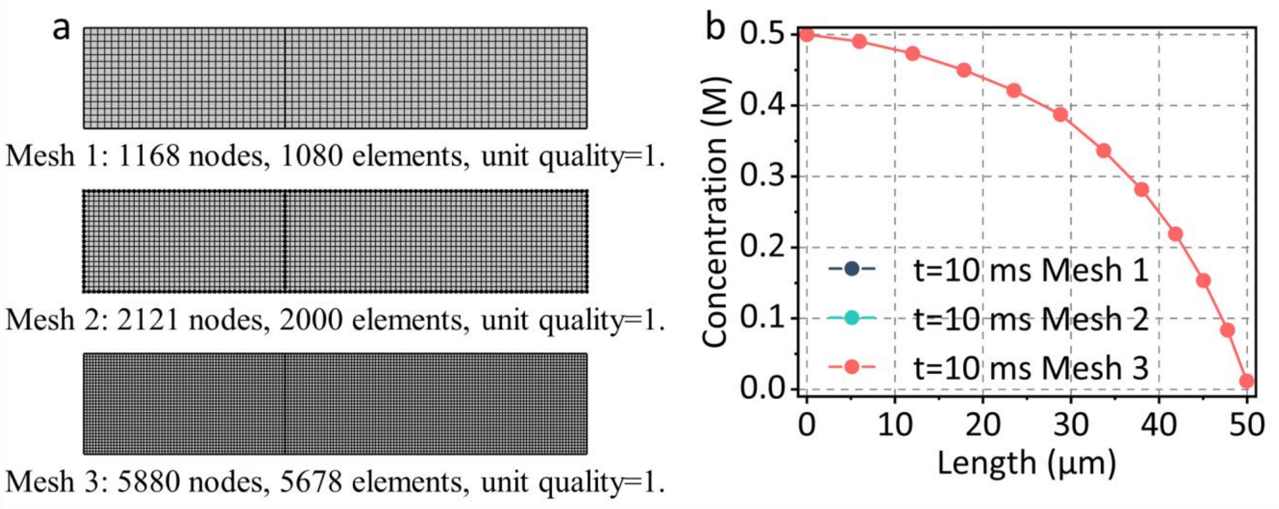

Figure S10. (a) Mesh configurations used for the MS-ZIF-A model and (b) concentration profiles along the channel at t = 10 ms obtained using different mesh densities.

Through the research, the visualized post-processing diagrams of the electric field mode, electric potential, concentration, and related data can be obtained. The distribution map of the electric field mode can help identify the regions where the electric field is concentrated, the distribution of the electric potential can help understand the distribution of charges and the force exerted by the electric field, and the concentration diagram shows the mass distribution of different substances in the system over time and space.

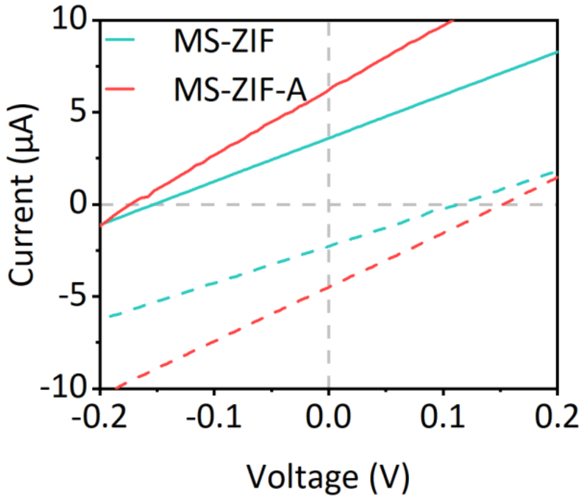

Figure S11. *I-V* test of the MS-ZIF and MS-ZIF-A membranes measured under two configurations by exchanging the placement of artificial seawater and river water between the two half-cells. Solid lines represent the configuration in which the high-concentration solution was placed on the MS side, while dashed lines represent the reverse configuration with the low-concentration solution on the MS side.

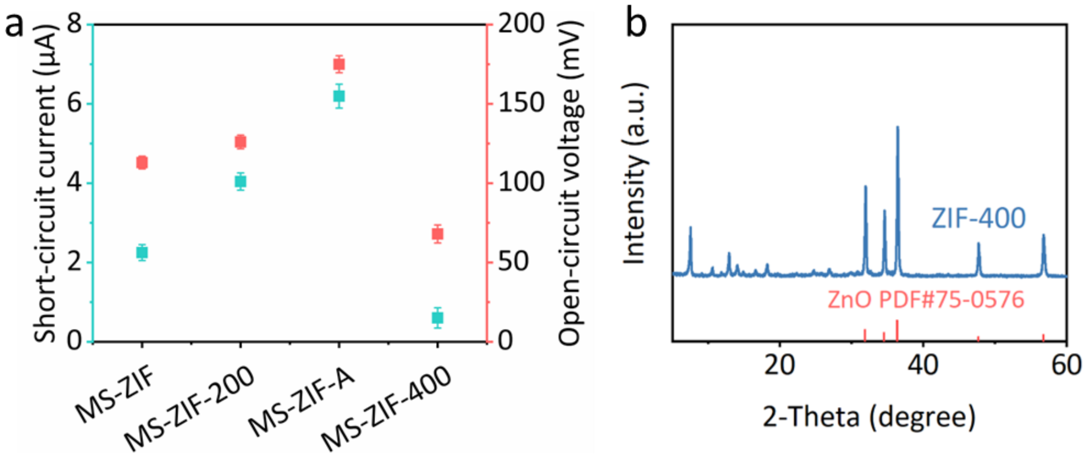

Figure S12. (a) I_{sc} and V_{oc} of MS-ZIF, MS-ZIF-200, MS-ZIF-A and MS-ZIF-400 measured under a 50-fold NaCl concentration gradient. (b) XRD pattern of ZIF-400.

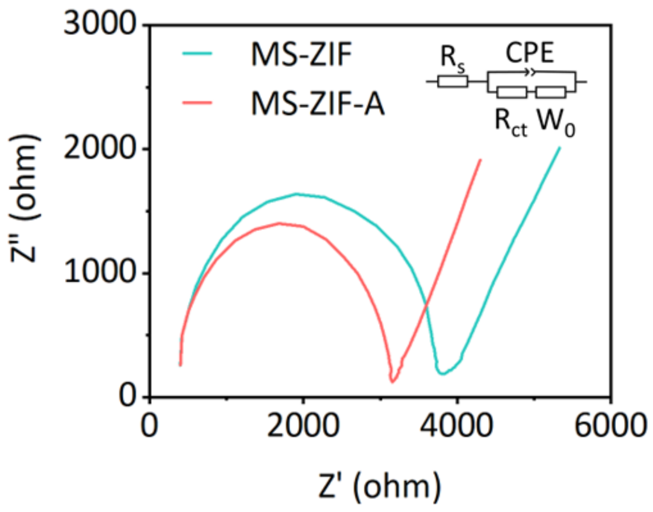

Figure S13. EIS spectra of the MS-ZIF and MS-ZIF-A membranes.

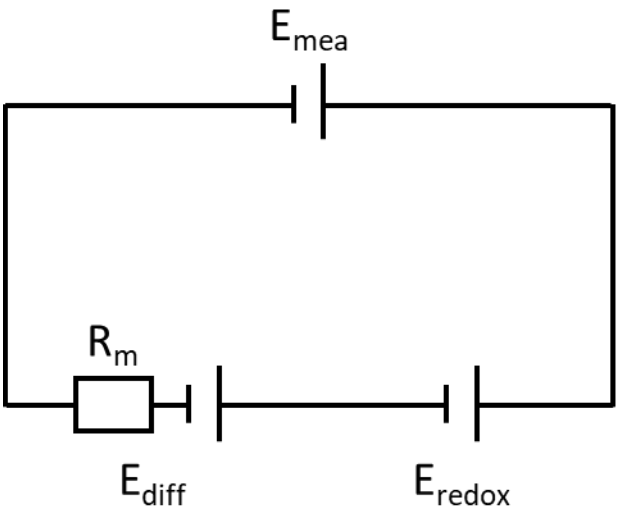

Figure S14. Schematic of the equivalent circuit used to analyse the salinity-gradient energy conversion system.

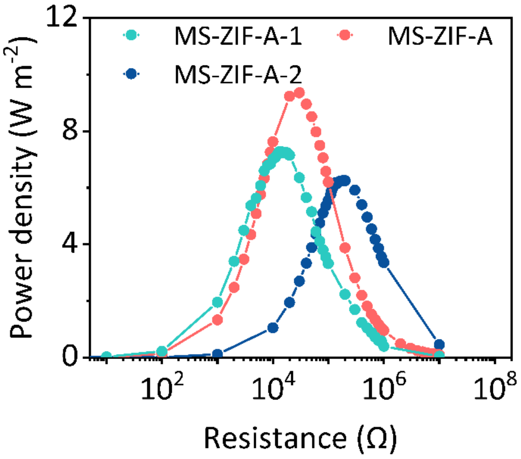

Figure S15. The power density of MS-ZIF-A-1, MS-ZIF-A and MS-ZIF-A-2 membranes at a 50-fold NaCl concentration.

Note: Membranes with varying MoS_2 loadings on ZIF-A were prepared by adjusting the MoS_2 content, yielding MS-ZIF-A-1 (MoS_2: 10 mg), MS-ZIF-A (MoS_2:20 mg) and MS-ZIF-A-2 (MoS_2:30 mg). As shown in Figure S15, the MS-ZIF-A membrane exhibited the highest output power density and the lowest transport resistance, confirming that the MS-ZIF-Awas optimal.

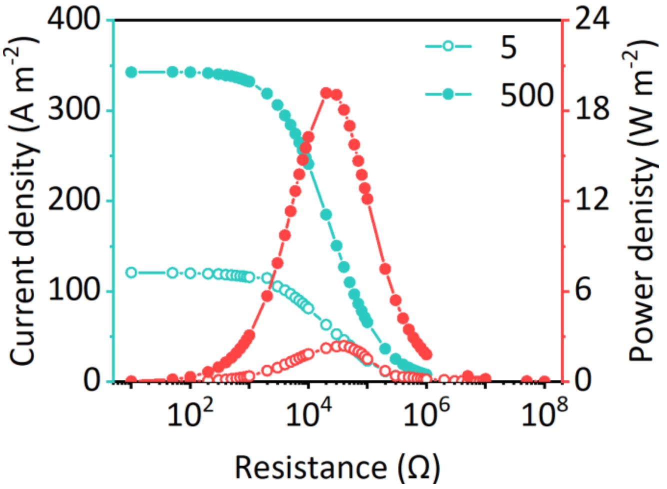

Figure S16. Current density and power density generated by the MS-ZIF-A membrane under 5- and 500-fold concentration gradients, with the low concentration reservoir fixed at 0.01 M.

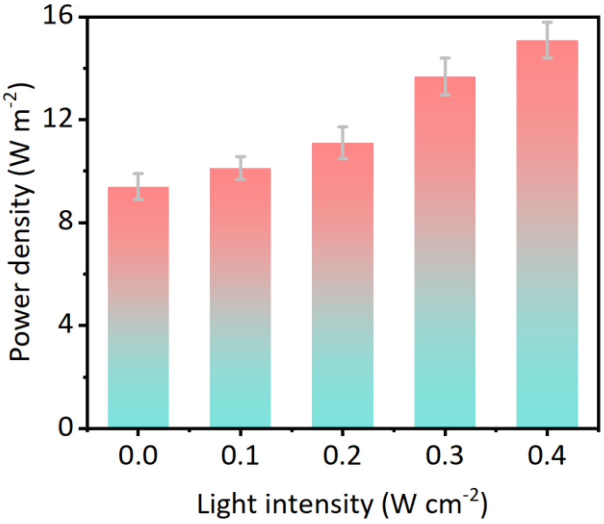

Figure S17. Osmotic energy conversion performance of the MS-ZIF-A membrane measured under different light intensities. Error bars represent mean ± SD, n=3.

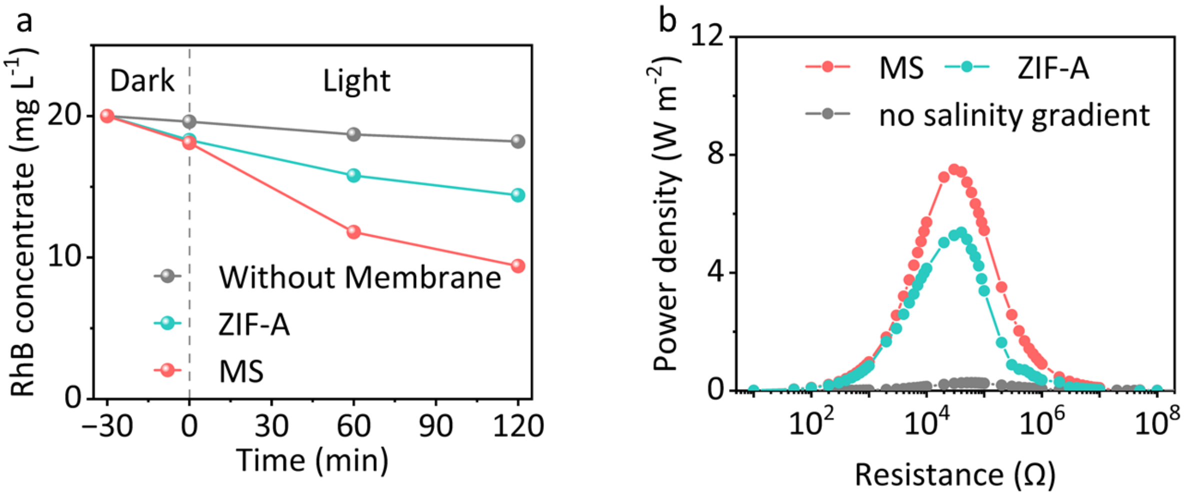

Figure S18. (a) Photocatalytic degradation of RhB by MS, ZIF-A and without membrane under visible light. (b) Osmotic power output of MS and ZIF in 0.5 M NaCl solution with RhB and MS-ZIF-A in no salinity gradient.

Note: As shown in Figure S18a, RhB adsorption on the as-prepared membrane was negligible in the dark. In the absence of a membrane, only 9.1% of RhB was degraded under illumination, confirming that direct photolysis made only a minor contribution. Moreover, the MoS_2 membrane exhibited higher photocatalytic activity toward RhB degradation than the ZIF-A membrane, indicating that MoS_2 served as the primary photocatalysis component. The role of the salinity gradient and asymmetric heterostructure in power generation was further examined. Under zero salinity-gradient conditions, the MS-ZIF-A membrane produced only a very low power density of 0.3 W m^{-2}, demonstrating that the salinity gradient is essential for osmotic energy conversion. Under identical salinity-gradient conditions, the MS and ZIF-A membranes delivered power densities of 7.5 and 5.4 W m^{-2}, respectively, both lower than that of the MS-ZIF-A membrane (11.1 W m^{-2}, Figure S18b). These results indicate that the synergistic coupling between the MoS_2 and ZIF-A layers, together with the salinity gradient, is crucial for achieving enhanced osmotic energy conversion.

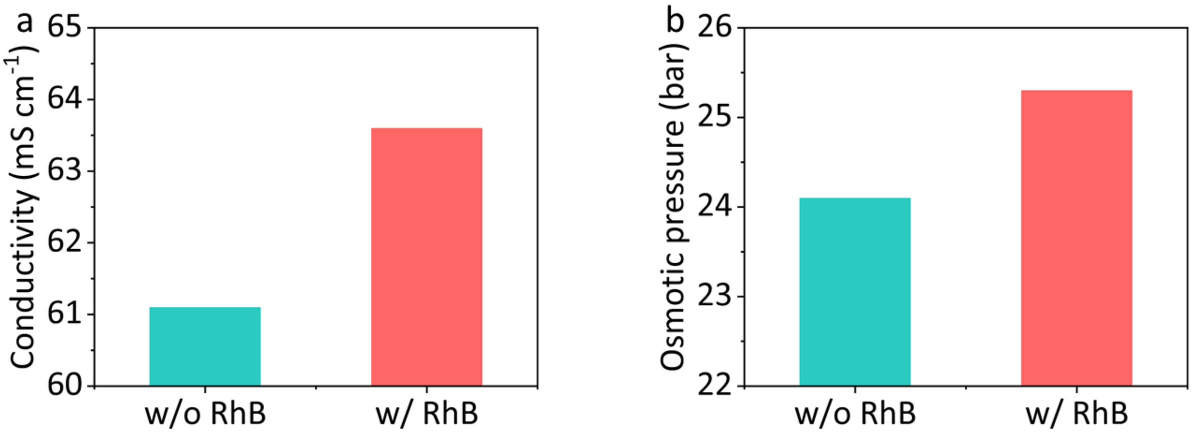

Figure S19. (a) The electrical conductivity and (b) osmotic pressure of a 0.5 M NaCl solution with and without 20 mg L^{-1} RhB.

Note: The comparison of electric conductivity between NaCl solutions with and without RhB showed only a negligible difference (Figure S19a), whereas the osmotic pressure increased slight after RhB addition (Figure S19b).

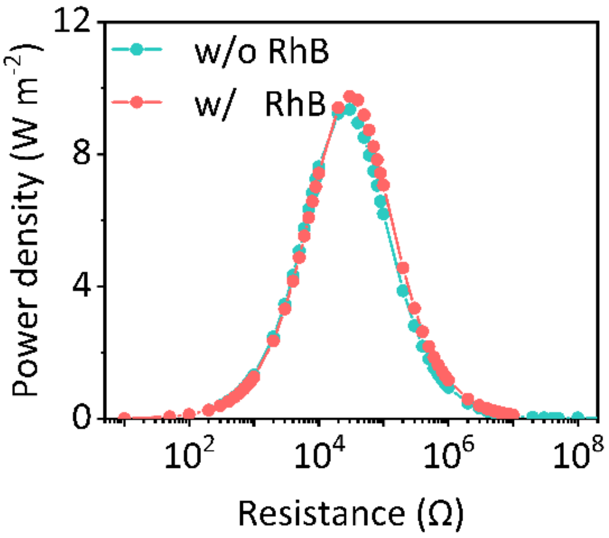

Figure S20. Osmotic power output of MS-ZIF-A in 0.5 M NaCl solution with and without 20 mg mL^{-1} RhB.

Note: We performed a control experiment under identical salinity gradient (0.5 M NaCl vs. 0.01 M NaCl) without RhB. The measured power density was 9.36 W m^{-2} (without RhB) vs. 9.75 W m^{-2} (with 20 mg L^{-1} RhB) under illumination. This confirm that RhB slightly enhances power output, but the core performance of the membrane does not rely on the presence of RhB.

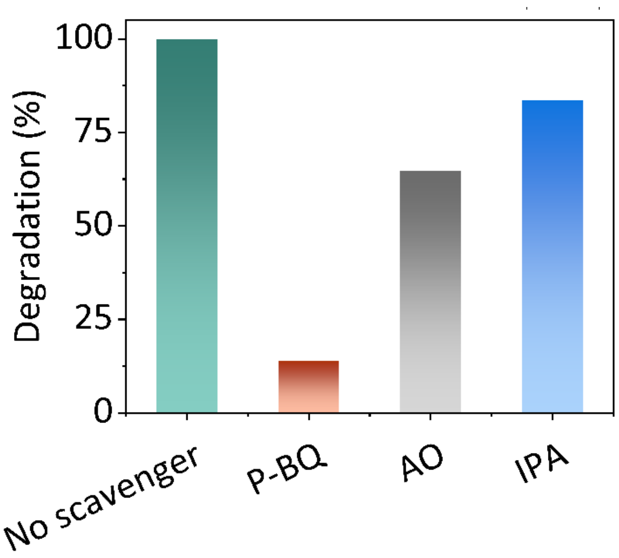

Figure S21. Degradation efficiency of RhB under different quenching conditions.

Note: The effect of different quenchers on the photocatalytic degradation efficiency of RhB by MS-ZIF-A was evaluated (Figure S21). Upon addition of p-benzoquinone (p-BQ), a superoxide radical ($\bullet O_2^-$) scavenger, the degradation efficiency was drastically reduced from 99.85% to 13.96%. When ammonium oxalate (AO), a hole (h^+) scavenger, and isopropanol (IPA), a hydroxyl radical (•OH) scavenger, were introduced, the efficiencies were reduced to 64.81% and 83.65%, respectively, by which a significant contribution of h^+ was confirmed. It was therefore indicated that a major role was played by $\bullet O_2^-$ in the degradation process, while a relatively minor role was played by •OH.

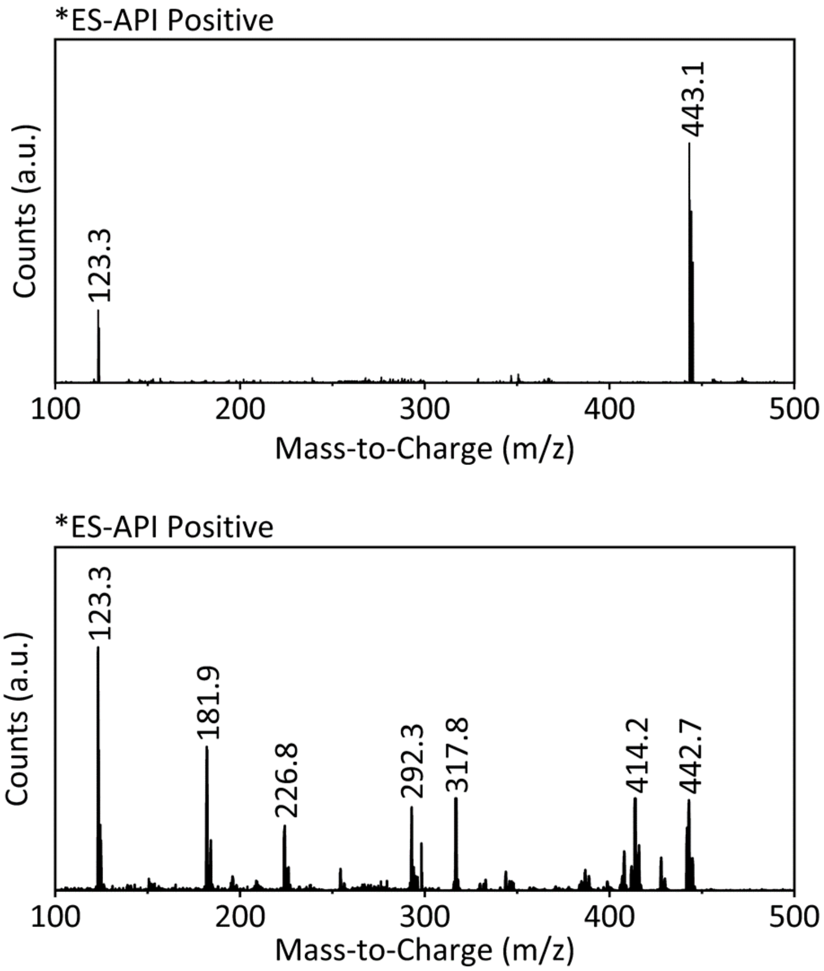

Figure S22. LC-MS spectra of the intermediate products from the photocatalytic degradation of RhB by MS-ZIF-A.

Note: The degradation solutions before and after irradiation were analyzed by liquid chromatography-mass spectrometry (LC-MS). In the mass spectrum of Figure S16, a series of m/z signals were observed at 443, 415, 318, 292, 123, 299, 227, and 182. These signals were attributed to RhB intermediates generated by the oxidation of holes (h^+) and superoxide radicals ($\bullet O_2^-$). This finding was consistent with previous reports. Additionally, a TOC degradation efficiency of 52.3% was achieved for RhB by MS-ZIF-A. It was thus inferred that, under prolonged attack by these reactive species, all intermediates were oxidized into small molecules and fully mineralized to carbon dioxide and water.

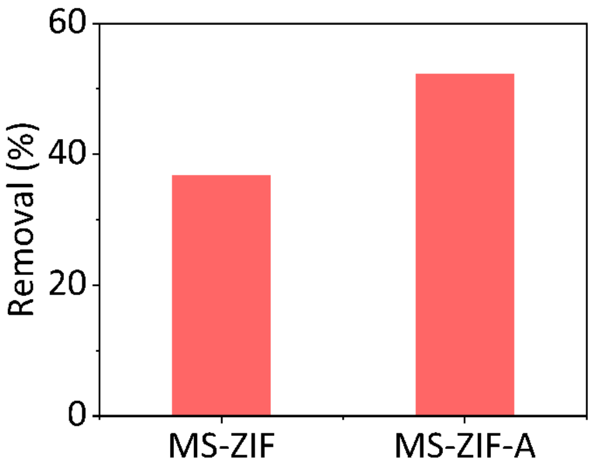

Figure S23. Removal of total organic carbon by photocatalytic degradation of RhB under visible light irradiation.

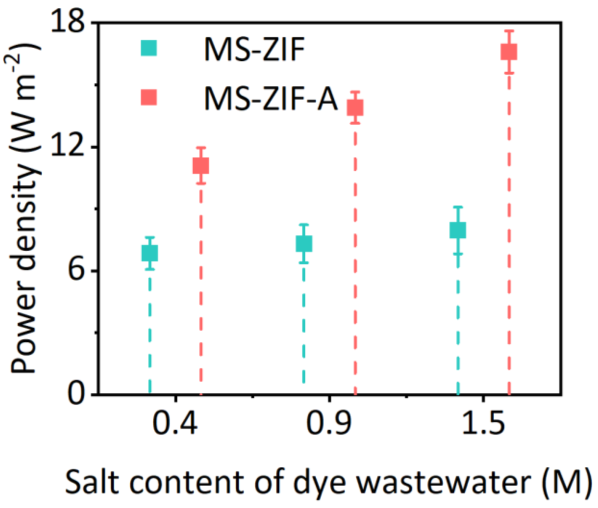

Figure S24. Power densities of MS-ZIF and MS-ZIF-A membranes measured in RhB-containing wastewater with different NaCl concentrations. Error bars represent mean ± SD, n=3.

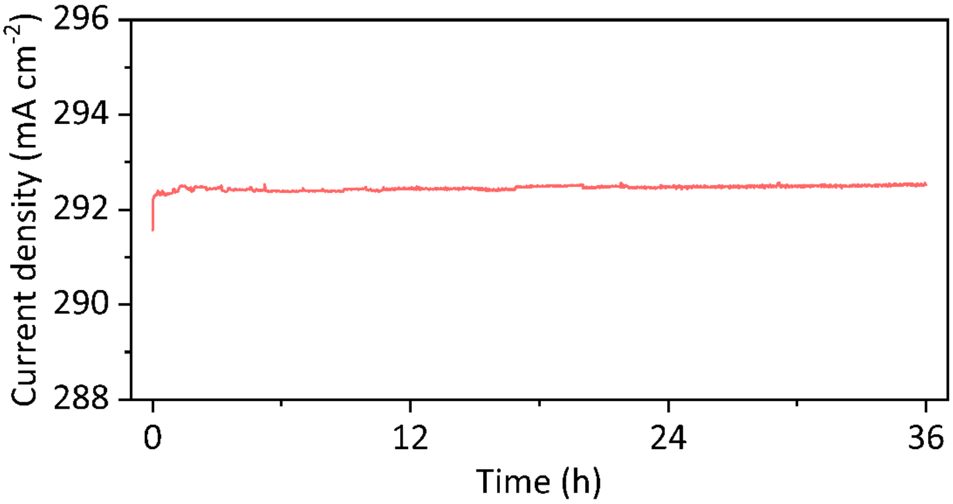

Figure S25. I-T curve of MS-ZIF-A membrane for 36 h with extra resistance of 20 kΩ.

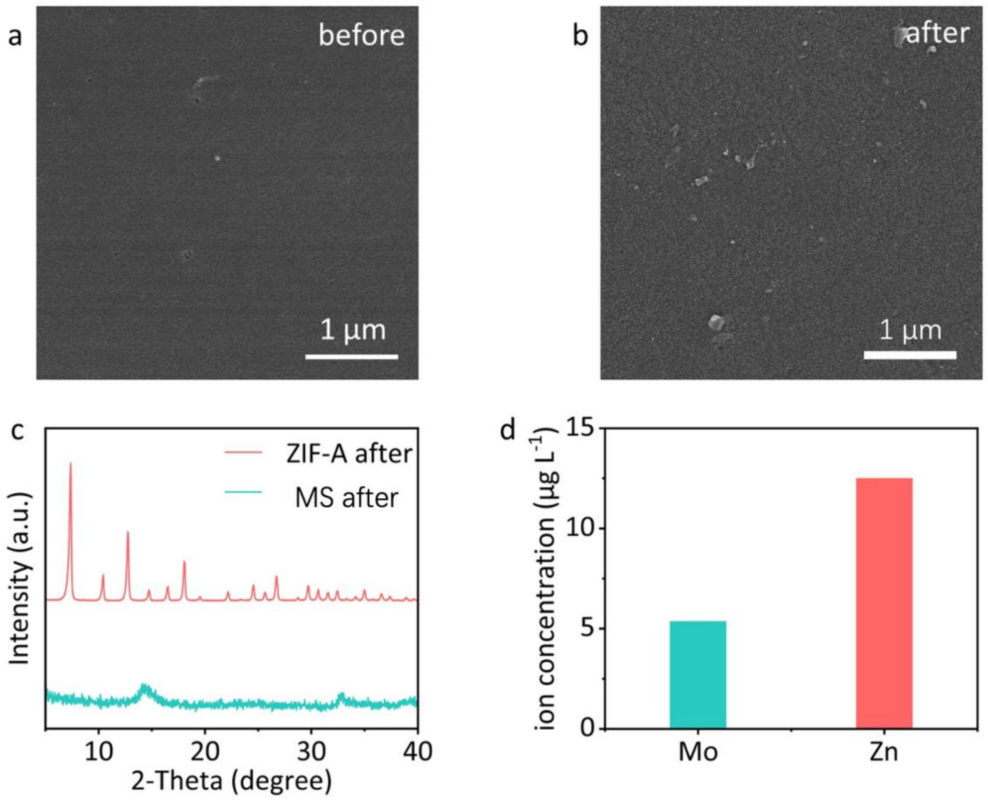

Figure S26. (a, b) SEM images, (c) XRD pattern, and (d) concentration of Mo and Zn ions dissolved in solution after continuous operation of MS-ZIF-A for 36 h.

Table S1. List of E_{mea}, E_{redox} and E_{diff} for MS-ZIF-A membranes, with the low-concentration reservoir fixed at 0.1 mM KCl.

E (mV)	10-fold	100-fold	1000-fold	3000-fold
E_{mea}	53	114	147	161
E_{redox}	23	54	74	86
E_{diff}	30	60	73	75

Table S2. Calculated t_+ and η of MS-ZIF-A under different concentration gradients.

Concentration	10-fold	100-fold	1000-fold	3000-fold
t_+	0.90	0.85	0.78	0.77
η (%)	32.3	24.0	16.0	15.1

Table S3. Comparison of the peak output power density and corresponding external load resistance of the MS-ZIF-A membrane with reported state-of-the-art asymmetric membranes.

Number	Membrane	Electrolyte	salinity gradient	Test area (mm^2)	External resistance load (kΩ)	Power density ($W\ m^{-2}$)	Ref.
1	MS-ZIF-A	NaCl	0.5 M/0.01M	0.03	30	9.4	This work
2	$Ni_2(OH)_2$@AAO	KCl	0.5 M/0.01M	0.03	12.21	5.05	[7]
3	GO/2DMC	NaCl	0.5 M/0.01M	0.03	5	6.41	[8]
4	PA-GO/AAO	KCl	0.5 M/0.01 M	0.03	10.5	3.73	[9]
5	UiO-66-NH_2@ANM	KBr	1 M/0.01 M	0.03	30	4.93	[10]
6	Nafion/ANM	NaCl	0.5 M/0.01 M	0.03	24	5.13	[11]
7	MXene/BNC	NaCl	0.5 M/0.01 M	1	40	0.91	[12]
8	MXene/PS-b-P2VP	NaCl	0.5 M/0.01 M	0.03	5.5	6.74	[13]
9	ZGDHM	NaCl	0.5 M/0.01 M	0.03	32	5.44	[14]
10	MCS/AAO	NaCl	0.5 M/0.01 M	0.03	10	5.04	[15]
11	ANF/Gel	NaCl	0.5 M/0.01 M	0.03	23	3.9	[16]
12	HENM	NaCl	0.5 M/0.01 M	0.03	15	2.2	[17]
13	CS/SA hydrogel	KCl	0.5 M/0.01 M	0.03	8	7.86	[18]
14	BHMXM	NaCl	0.5 M/0.01 M	0.03	10	8.6	[19]
15	P-MOF-AAO	KCl	3 M/0.01 M	0.13	20	6.26	[20]
16	CMWS	NaCl	0.5 M/0.01 M	0.03	13	2.78	[21]
17	CMS/AAO	NaCl	0.5 M/0.01 M	0.03	13	3.25	[22]

References

1. Li, Y. S.; Liang, F. Y.; Bux, H.; Feldhoff, A.; Yang, W. S.; Caro, J., *Angewandte Chemie International Edition* **2010,** *49* (3), 548-551.
2. Xiong, F.; Hu, H.; Xue, X.; Wu, M.; Zhou, J.; Zhang, W.; Li, R., *Water Research* **2024,** *259*, 121888.
3. Ouyang, W.; Wang, W.; Zhang, H.; Wu, W.; Li, Z., *Nanotechnology* **2013,** *24* (34), 345401.
4. Feng, J.; Graf, M.; Liu, K.; Ovchinnikov, D.; Dumcenco, D.; Heiranian, M.; Nandigana, V.; Aluru, N. R.; Kis, A.; Radenovic, A., *Nature* **2016,** *536* (7615), 197-200.
5. Xin, W.; Zhang, Z.; Huang, X.; Hu, Y.; Zhou, T.; Zhu, C.; Kong, X.-Y.; Jiang, L.; Wen, L., *Nature Communications* **2019,** *10* (1), 3876.
6. Zhang, Z.; Yang, S.; Zhang, P.; Zhang, J.; Chen, G.; Feng, X., *Nature Communications* **2019,** *10* (1), 2920.
7. Zeng, H.; Yao, C.; Wu, C.; Wang, D.; Ma, W.; Wang, J., *Small* **2024,** *20* (26), 2310811.
8. He, Y.; Huang, Z.; Xie, L.; Zhang, X.; Hu, X.; Liang, K.; Jiang, L.; Zhou, S.; Kong, B., *Small* **2023,** *20* (11), 2306910.
9. Zhang, L.; Zhou, S.; Xie, L.; Wen, L.; Tang, J.; Liang, K.; Kong, X.; Zeng, J.; Zhang, R.; Liu, J.; Qiu, B.; Jiang, L.; Kong, B., *Small* **2021,** *17* (13), 2100141.
10. Liu, Y. C.; Yeh, L. H.; Zheng, M. J.; Wu, K. C. W., *Science Advances* *7* (10), eabe9924.
11. Chang, C.-W.; Chu, C.-W.; Su, Y.-S.; Yeh, L.-H., *Journal of Materials Chemistry A* **2022,** *10* (6), 2867-2875.
12. Xu, Y.; Zhang, K.; Chen, S.; Zhang, X.; Chen, Y.; Li, D.; Xu, F., *Electrochimica Acta* **2022,** *412*, 140162.
13. Lin, X.; Liu, P.; Xin, W.; Teng, Y.; Chen, J.; Wu, Y.; Zhao, Y.; Kong, X. Y.; Jiang, L.; Wen, L., *Advanced Functional Materials* **2021,** *31* (45), 2105013.
14. Huang, K. T.; Hung, W. H.; Su, Y. C.; Tang, F. C.; Linh, L. D.; Huang, C. J.; Yeh, L. H., *Advanced Functional Materials* **2023,** *33* (19), 2211316.
15. Zhou, S.; Xie, L.; Li, X.; Huang, Y.; Zhang, L.; Liang, Q.; Yan, M.; Zeng, J.; Qiu, B.; Liu, T.; Tang, J.; Wen, L.; Jiang, L.; Kong, B., *Angewandte Chemie International Edition* **2021,** *60* (50), 26167-26176.
16. Zhang, Z.; He, L.; Zhu, C.; Qian, Y.; Wen, L.; Jiang, L., *Nature Communications* **2020,** *11* (1), 875.

17. Ling, H.; Xin, W.; Qian, Y.; He, X.; Yang, L.; Chen, W.; Wu, Y.; Du, H.; Liu, Y.; Kong, X. Y.; Jiang, L.; Wen, L., *Angewandte Chemie International Edition* **2023,** *62* (1), e202212120.

18. Bian, G.; Pan, N.; Luan, Z.; Sui, X.; Fan, W.; Xia, Y.; Sui, K.; Jiang, L., *Angewandte Chemie International Edition* **2021,** *60* (37), 20294-20300.

19. Ding, L.; Zheng, M.; Xiao, D.; Zhao, Z.; Xue, J.; Zhang, S.; Caro, J.; Wang, H., *Angewandte Chemie International Edition* **2022,** *61* (41), e202206152.

20. Li, Z. Q.; Zhu, G. L.; Mo, R. J.; Wu, M. Y.; Ding, X. L.; Huang, L. Q.; Wu, Z. Q.; Xia, X. H., *Angewandte Chemie International Edition* **2022,** *61* (22), e202202698.

21. Xie, L.; Zhou, S.; Liu, J.; Qiu, B.; Liu, T.; Liang, Q.; Zheng, X.; Li, B.; Zeng, J.; Yan, M.; He, Y.; Zhang, X.; Zeng, H.; Ma, D.; Chen, P.; Liang, K.; Jiang, L.; Wang, Y.; Zhao, D.; Kong, B., *Journal of the American Chemical Society* **2021,** *143* (18), 6922-6932.

22. Huang, Y.; Zeng, H.; Xie, L.; Gao, R.; Zhou, S.; Liang, Q.; Zhang, X.; Liang, K.; Jiang, L.; Kong, B., *Journal of the American Chemical Society* **2022,** *144* (30), 13794-13805.
